# Effect of the Hydrostatic High Pressures (HHP) on the Microbiological and Physicochemical Properties of *‘Picual’ cv.* and *‘Morisca’ cv.* Olive Pomaces

**DOI:** 10.3390/foods15193549

**Published:** 2026-10-05

**Authors:** Jesús J. García-Parra, María Cabeza de Vaca, Manuel A. Martínez-Cañas, Antonia M. Trejo

**Affiliations:** Technological Institute of Food and Agriculture (INTAEX), Center for Scientific and Technological Research of Extremadura (CICYTEX), Avda, Adolfo Suárez s/n, 06007 Badajoz, Spain; maria.cabeza@juntaex.es (M.C.d.V.); manuel.martinez@juntaex.es (M.A.M.-C.); antoniamaria.trejo@juntaex.es (A.M.T.)

**Keywords:** *Olea europaea* L., by-product upcycling, high-hydrostatic process, phenolic profile, hydroxytyrosol, polyphenol oxidase

## Abstract

Olive pomace (alperujo) is a major by-product of the virgin olive oil industry with a substantial environmental impact but remarkable potential as a health-promoting functional food ingredient. However, its high moisture and organic load necessitate immediate stabilization to prevent degradation. This study evaluated the efficacy of High Hydrostatic Pressure (HHP) processing (400 and 600 MPa for 5 min) as a non-thermal technology to stabilize *‘Picual’* and *‘Morisca’* olive pomaces harvested at two distinct ripening stages (green and ripe). HHP treatments achieved complete microbial inactivation, reducing aerobic mesophiles, molds, and yeasts below detection limits (<1.0 log CFU g^−1^) regardless of cultivar or ripeness, while exceptionally preserving the macroscopic color profile with total color differences (ΔE_HHP_) remaining at about or below the human visual perception threshold (<3.0). Conversely, endogenous polyphenol oxidase (PPO) exhibited significant baroresistance, remaining statistically unaltered and active after processing. The pressure-induced cellular disruption, combined with this residual PPO activity, triggered a selective oxidative cascade: ortho-diphenols (hydroxytyrosol, oleuropein, and the verbascoside+rutin fraction) significantly decreased, whereas monophenols (tyrosol and p-coumaric acid) and flavonoid glycosides remained highly stable throughout processing for *‘Morisca’* but exhibited reductions in *‘Picual’*, due to their lack of a reactive catechol moiety. Despite these slight oxidative changes, a strong linear correlation (*p* < 0.001) between total phenolic content and antioxidant activity was maintained. Furthermore, HHP preserved individual profiles of unsaturated fatty acids, such as oleic and linoleic acids. Multivariate Principal Component Analysis (PCA) confirmed that HHP effectively safeguards the macro-physicochemical and nutritional identity of the matrix while ensuring microbiological safety. These findings demonstrate that HHP is a robust bio-stabilization tool, particularly when applied to early-harvested (green) matrices, enabling the sustainable upcycling of olive pomace into safe, lipid-stable, and antioxidant-rich functional food ingredients.

## 1. Introduction

Olive oil is the cornerstone of the Mediterranean diet, globally acclaimed for its nutritional excellence, high concentration of unsaturated fatty acids, and potent antioxidant phenolic compounds [1]. Spain remains the undisputed global leader in olive oil production, yielding over one million tons annually. In Spain, the region of Extremadura plays a strategic role as a primary producing area, cultivating widespread cultivars such as *‘Picual’* alongside autochthonous cultivars of high regional value, such as *‘Morisca’* [2]. However, the modernization of the olive milling industry, which heavily relies on the continuous two-phase centrifugation system, inherently generates massive volumes of a semi-solid by-product known as olive pomace or wet olive pomace (locally known as *alperujo*) [3,4].

The production of olive pomace is highly localized and concentrated within a short seasonal window (November to March). Its high moisture content (around 60–70%), acidic pH, and heavy load of phenolic compounds render its management a critical environmental and economic bottleneck for the olive industry. In regions like Extremadura, the long-term storage of this waste in artificial lagoons poses severe soil and groundwater contamination risks [5,6]. Under the framework of a sustainable circular bioeconomy, there is an urgent paradigm shift towards valorizing this by-product rather than utilizing it merely for low-value applications such as combustion or composting [1].

Olive pomace is an exceptional, low-cost source of dietary fiber, residual unsaturated fatty acids (predominantly oleic acid), and highly bioactive polyphenols, notably hydroxytyrosol and oleuropein [7,8]. These phytochemical profiles make olive pomace an outstanding candidate for the recovery of natural antioxidants and the formulation of innovative, high-added-value functional foods [9,10]. While some residual biomedical applications have been explored, the primary and most viable translational potential of this biomass lies in the food industry, where it can act as a natural preservative and fortifying ingredient [11,12]. However, to obtain a long-term availability and adequate preservation, conventional thermal pasteurization methods often fail to preserve their functional quality, as high temperatures trigger the thermal degradation of thermolabile antioxidants and catalyze the auto-oxidation or isomerization of unsaturated lipids [8].

High Hydrostatic Pressure (HHP) has emerged as a disruptive, non-thermal preservation technology capable of achieving microbial inactivation at room temperature while preserving low-molecular-weight compounds [13]. Previous studies have demonstrated the efficacy of HHP in preserving olive-derived products, such as dressed table olives and olive jams [14,15], but its application for the direct stabilization of raw olive pomace for food applications remains underexplored. Unlike processed olive-derived products, raw alperujo is a highly reactive and enzymatically unstable matrix [3,8]. Therefore, understanding how the genetic background and endogenous enzymatic baroresistance limit the recovery of bioactive compounds under HHP conditions represents an important and significant step towards their industrial utilization. Crucially, it is unknown how inherent biological factors—such as cultivar genetics and the fruit’s ontogenetic (ripening) stage—condition the matrix’s technological response to HHP treatments. Fruit maturation drastically alters the balance between endogenous oxidative enzymes, such as polyphenol oxidase (PPO), and their phenolic substrates [16,17]. However, how this complex biochemical background interacts with high pressure has not been addressed.

To address this gap, the present study establishes a comprehensive evaluation of non-thermal stabilization by HHP treatments (up to 600 MPa) on the microbiological safety, chromatic parameters, and phytochemical profiles of fresh olive pomace from two distinct cultivars, the widely cultivated *‘Picual’* and the Extremadura autochthonous *‘Morisca’*, harvested at two different ripening stages. It was hypothesized that HHP processing would serve as an effective method for both microbial and enzymatic inactivation, and that the technological viability of the treatment would be dictated by the balance between pathogen destruction and bioactive compound retention across different matrices. Therefore, the main objective is to establish the technological viability of HHP treatments as an effective method for both microbial and enzymatic inactivation, enabling the optimal retention of bioactive compounds and facilitating the sustainable upcycling of this raw material as a high-value by-product. Ultimately, this will establish a robust foundation for the sustainable integration of stabilized olive pomace as a safe, functional ingredient in the food industry.

## 2. Materials and Methods

### 2.1. Cultivar Selection and Olive Pomace Obtention

Fresh olive fruits (*Olea europaea* L.) from two representative cultivars of the Extremadura region, *‘Morisca’* and *‘Picual’*, were harvested at two distinct ripening stages: green (Ripeness Index, MI ≈ 1.5) and ripe (MI ≈ 4.5), determined according to the method described by Uceda and Frias [18], by a local olive mill industry (Cooperative Virgen de la Estrella, Los Santos de Maimona, Extremadura, Spain). The fruits were characterized (Ripeness index, health status, and physical characteristics, Table 1) and processed immediately after harvest using a standardized industrial three-phase extraction system (Abencor MC2, Seville, Spain) located at CICYTEX (Badajoz, Spain) to obtain the wet olive pomace. This process resulted in four types of olive pomace: *Morisca* green (MG), *Morisca* ripe (MM), *Picual* green (PG), and *Picual* ripe (PR). Byproducts were promptly vacuum-sealed in polyethylene bags and stored at 4 °C in the absence of light until processing [19].

### 2.2. Hydrostatic High Pressure (HHP) Stabilization Treatment

The HHP treatments were performed using a pilot-scale hydrostatic pressure system (Hiperbaric Wave 6000/55, Hiperbaric, Burgos, Spain), characterized by a 55 L capacity chamber and a maximum operational threshold of 600 MPa. Two pressure levels were applied: 400 MPa and 600 MPa, both applied for a dwell time of 5 min at an initial water temperature of 14 °C [14]. The pressure-transmitting medium was water, and the compression rate was maintained at approximately 200 MPa/min. Decompression was nearly instantaneous (<3 s). Non-treated samples served as the experimental control for each cultivar and ripening stage.

Each treatment batch consisted of 5 independent replicates (vacuum-packaged portions). Therefore, the experimental design resulted in a total of 60 independent samples (2 varieties × 2 maturity stages × 3 HHP treatments × 5 replicates). Furthermore, all subsequent physicochemical and microbiological determinations were performed using 5 replicates for each sample.

After processing, the samples were stored at 4 °C in the absence of light. Microbiological analysis and color determinations were performed the day after the HHP treatments on the fresh olive pomace samples. The remaining olive pomace was frozen at −80 °C until further analysis.

### 2.3. Microbiological Analysis

In conformity with EU normative [20], microbiological assays were performed according to ISO standards. Ten grams of fresh olive pomace were homogenized in 90 mL of sterile peptone water (0.1% *w*/*v*). Total aerobic mesophilic bacteria were quantified on Plate Count Agar (PCA) after incubation at 30 °C for 72 h (ISO 4833-1:2013) [21]. Total molds and yeasts were determined on yeast glucose chloramphenicol (YGC) agar at 25 °C for 5 days (ISO 21527-2:2008) [22]. Enterobacteriaceae counts were monitored on Violet Red Bile Glucose (VRBG) (ISO 21528-1:2017) [23]. Results were expressed as the log of colony-forming units per gram of fresh olive pomace (CFU g^−1^).

### 2.4. Instrumental Color and Chlorophyll Analysis

The chromatic profile of the olive pomace was determined using a spectrophotocolorimeter (Minolta CM-508i) in the CIELAB color space. The parameters L* (lightness), a* (redness/greenness), and b* (yellowness/blueness) were recorded. From these coordinates, the total color difference (ΔE) was calculated to assess the perceptibility of pressure-induced shifts.

$${\Delta E}_{\mathrm{HHP}}=\sqrt{{\left({L^{*}-L^{*}}_{\mathrm{control}}\right)}^{2}{+\left({a^{*}-a^{*}}_{\mathrm{control}}\right)}^{2}{+\left({b^{*}-b^{*}}_{\mathrm{control}}\right)}^{2}}$$

The quantification of total chlorophyll was determined spectrophotometrically [24]. For the extraction of chlorophylls, 7 mL of acetone was added to 3.5 g of thawed olive pomace and homogenized for 1 min using a T18 digital ULTRA-TURRAX (IKA-Werke GmbH, Staufen, Germany) under cool conditions. The samples were then placed in an ultrasound bath (J.P. SELECTA, Barcelona, Spain) for 10 min and subsequently centrifuged at 3000 rpm for 5 min at 4 °C using a 5920R centrifuge (Eppendorf, Hamburg, Germany). The supernatant was carefully collected and filtered using 1300/80 filters (Filter lab, Filtros Ainoa, Barcelona, Spain). The remaining pellet was washed two additional times under the same conditions. The combined supernatants were adjusted to a final volume of 25 mL with acetone. The absorbance of each extract was measured at 646 and 661 nm, and the contents of both chlorophylls were calculated spectrophotometrically using equations derived from the partial least squares (PLS) model:

Chlorophyll a (µg/mL) = −2.7816 × Abs_646_ + 12.4228 × Abs_661_ − 0.0223

Chlorophyll b (µg/mL) = 16.7322 × Abs_646_ − 4.0583 × Abs_661_ − 0.0104

The results were expressed as µg of chlorophyll a and chlorophyll b per g of fresh olive pomace (µg g^−1^).

### 2.5. Characterizations of Bioactive Ingredients

#### 2.5.1. Obtention of Olive Pomace Extracts

The recovery of the phenolic extracts was carried out by macerating 2.5 g of olive pomace in 22.5 mL of ethanol, acidified with 1% (*w*/*v*) citric acid, under different conditions depending on the ripeness of fresh fruit, and according to optimal conditions previously standardized by our research group through a 3-factor response surface methodology (RSM). In the case of the green samples, the olive pomaces were macerated with 72% (*v*/*v*) ethanol at 69 °C for 82 min, whereas ripe olive pomaces at 82% (*v*/*v*) ethanol at 60 °C for 80 min. The resulting extracts were centrifuged (4 °C, 15 min), filtered, concentrated via rotary evaporation to remove the ethanol, and adjusted to 25 mL with distilled water before freezing at −80 °C.

#### 2.5.2. Total Phenolic Content (TPC) and Total Antioxidant Activity (TAA)

The TPC and TAA of the olive pomace extracts were quantified spectrophotometrically using a Tecan Spark microplate reader (Tecan Trading AG, Grödig, Austria). TPC was determined by the Folin–Ciocalteu assay [25]. In particular, 100 µL of extract was mixed with 1.6 mL of water and 100 µL of Folin–Ciocalteu reagent, followed by 200 µL of 30% (*w*/*v*) calcium carbonate. After 1 h incubation in darkness, absorbance was measured at 760 nm. Results were expressed as mg gallic acid equivalents (GAE) g^−1^ fresh olive pomace. TAA was measured via the ABTS^+^ method [26]. Specifically, 10 µL of extract was added to 200 µL of ABTS^+^ solution (7 mM, Abs 1.5 at 734 nm) and monitored for 30 min. Results were expressed as µmol Trolox equivalents (TE) g^−1^ fresh olive pomace.

#### 2.5.3. Phenolic Compounds Profile

The identification and quantification of individual phenolic compounds in olive pomace extracts were conducted using an Agilent 1200 high-performance liquid chromatography (HPLC) system equipped with a UV-Vis diode array detector (DAD) and a fluorescence spectrophotometric detector (FLD). Chromatographic separation was achieved on a reversed-phase Licrospher^®^ 100 RP-18 column (250 × 4.0 mm; 5 µm particle size) equipped with a pre-column. Operating conditions were as follows: column temperature, 40 °C; injection volume, 20 μL. The mobile phase was pumped at a constant flow rate of 1 mL min^−1^. In the mobile phase, two solvents were used in a gradient elution: phase A consisted of 0.1% formic acid in water, while phase B comprised 0.1% formic acid in acetonitrile. The following gradient was used: 0–1 min. 97% of A; 1–30 min. from 97% to 65% of A; 30–33 min. from 65% to 50% of A; 33–34 min. from 50% to 100% of A. Compound identification was performed through chromatographic co-elution (matching retention times) and spectral comparison with commercial standards. The reference standards utilized included hydroxytyrosol, tyrosol, oleuropein, verbascoside, rutin, luteolin-7-O-glucoside, apigenin-7-O-glucoside, and *p*-coumaric acid (Sigma-Aldrich, St. Louis, MO, USA, and Extrasynthese, Genay, France).

Hydroxytyrosol and tyrosol were identified and quantified using the FLD (excitation at 275 nm, emission at 315 nm), while the secoiridoid oleuropein was monitored by DAD at 255 nm. Additional DAD operational wavelengths included 320 nm for verbascoside and *p*-coumaric acid, and 350 nm for flavonoid glycosides such as apigenin-7-O-glucoside, luteolin-7-O-glucoside, and rutin. Quantification was executed using external calibration curves constructed from the corresponding specific standards, with results expressed as μg mL^−1^ of extract. Representative HPLC-DAD and HPLC-FLD chromatograms illustrating the phenolic profile are provided in the Supplementary Materials (Figures S1 and S2) [7,27].

#### 2.5.4. Fatty Acid Composition

Profile of fatty acids of olive pomaces was determined by fatty acid methyl esters (FAMEs), via GC-FID (Agilent 6890 model, Agilent Technologies, Palo Alto, CA, USA), and using a DB-23 column (60 m × 0.25 mm × 0.5 µm, Agilent Technologies, Palo Alto, CA, USA). Methylation was performed by diluting extracted fat 1:20 (*w*/*v*) in hexane and adding 50 µL of 2 M KOH/methanol per mL of solvent. The chromatographic conditions were set as follows: Helium was used as the carrier gas at a constant flow rate of 1.0 mL/min. The injection volume was 1 µL with a split ratio of 1:50. The injector and detector (FID) temperatures were both maintained at 250 °C. The oven temperature program was initialized at 160 °C for 1 min, then ramped at a rate of 4 °C/min to 210 °C and held at this final temperature for 10 min to ensure complete elution. Individual fatty acids were identified by comparing their chromatographic retention times (co-elution) with a commercial standard mixture (Supelco 37 Component FAME Mix, Sigma-Aldrich, St. Louis, MO, USA). Results were expressed as a relative percentage of total FAMEs. Representative GC-FID chromatograms illustrating the fatty acid profile are provided in the Supplementary Materials (Figures S3 and S4) [8].

### 2.6. Polyphenol Oxidase (PPO) Enzymatic Activity

The residual activity of PPO was measured spectrophotometrically following previously described methodologies for olive matrices [28]. Crude extracts were obtained using a phosphate buffer (pH 6.5) containing 1% (*w*/*v*) polyvinylpyrrolidone (PVPP). The assay monitored the increase in absorbance at 420 nm using catechol as a substrate at 25 °C. Results were expressed as the increase in the absorbance per minute (Δabs/min) under the conditions of the present assay.

### 2.7. Statistical Analysis

All analytical determinations were performed using five replicates, and the results are expressed as mean values ± standard deviation. Statistical analyses and multivariate modeling were conducted using SPSS software (v.25.0, IBM Corp., Armonk, NY, USA). To assess the impact of the experimental factors within each cultivar, a two-way analysis of variance (ANOVA) was utilized to determine the significance of the Ripening stage (R), High Hydrostatic Pressure treatment (P), and their interaction (R × P), with differences considered significant at *p* ≤ 0.05, *p* ≤ 0.01, and *p* ≤ 0.001. Furthermore, a one-way ANOVA followed by Tukey’s post hoc significant difference (HSD) test (*p* < 0.05) was applied to compare the specific pressure levels (Control, 400 MPa, and 600 MPa) within each independent ontogenetic stage. Additionally, two Principal Component Analysis (PCA) models were constructed: the first based on physicochemical and nutritional parameters, and the second integrating the microbiological counts to evaluate the overall technological efficacy of the treatments. Finally, a Partial Least Squares (PLS) regression model was developed to formulate predictive equations for the precise quantification of chlorophylls.

## 3. Results and Discussion

### 3.1. Effects of Ripeness and HHP on Morisca and Picual Olive Pomace Characteristics

Table 2 and Table 3 show the effect of the maturation and the HHP treatment on the microbiological loads, color, chemical composition, and phenolic compounds profile of the *‘Morisca’* and *‘Picual’* olive pomaces, as well as the interaction between both factors.

For both cultivars, the ripeness stage and HHP showed a remarkable effect on almost all the studied parameters, though some differences were found between cultivars. Specifically, for ‘*Morisca’ olive* pomaces, the ripeness did not affect the microbial loads. However, the color parameters and most of the chemical compounds were affected by ripeness. Only the Margaric acid (C17:0), Luteolin-7-O-glucoside, and Quercetin-3-O-rutinoside were not affected by the ripeness of the *‘Morisca’* cultivar.

The application of HHP significantly affected microbial loads and color parameters, but not luminosity and ΔE. The TAA, Myristic (C14:0), Palmitic (C16:0), Palmitoleic (C16:1), Margaric (C17:0), Stearic (C18:0), Gadoleic (C20:1) acids, trans-ferulic acid, apigenin-7-O-glucoside, luteolin-7-O-glucoside, Oleuropein, and p-Coumaric acid were also affected by HHP. On the other hand, in the ‘*Picual’ olive* pomaces, ripeness affected the mesophiles and mold and yeast loads, the color parameters, and also all the chemical parameters, but Margaric acid (C17:0), araquic acid (C20:0), and Hydroxytyrosol. Similarly, almost all the studied parameters were affected by HHP, but the a*, hue, PPO activity, lauric (C12:0), margaric (C17:0), cis-9-oleic (C18:1), linoleic (C18:2. cis.cis), and linolenic (C18:3) acids, as well as trans-ferulic acid, apigenin-7-O-glucoside, hydroxytyrosol, luteolin-7-O-glucoside, and oleuropein phenols. Thus, according to our results, the ripeness stage of olive fruit and the application of HHP treatments are two factors to consider in the final quality of the olive pomaces.

### 3.2. Effects of HHP on Microbial Loads

The microbial quality of fresh olive pomace is a fundamental parameter for its valorization as a functional food ingredient, since its high moisture and organic contents can promote rapid degradation by microbiological spoilage. The initial microbial populations of the untreated control samples, as shown in Table 4, revealed high counts of aerobic mesophilic bacteria (ranging from 4.82 to 6.15 log CFU g^−1^) and molds and yeasts (4.54 to 5.82 log CFU g^−1^). These baseline values are consistent with the typical microbiota found in two- and three-phase olive mill by-products, where the presence of mesophilic bacteria often dominates the initial stages of spoilage [29,30].

The application of HHP treatments at 400 and 600 MPa (5 min) achieved a complete reduction in the microbial load. According to the data summarized in Table 4, both pressure levels were sufficient to reduce aerobic mesophiles, molds, and yeasts to levels near 2 log CFU g^−1^, or even below the detection limit (<1.0 log CFU g^−1^), regardless of the cultivar (*‘Morisca’* or *‘Picual’*) or the ripeness stage. This drastic inactivation confirms the efficacy of HHP as a non-thermal bio-sanitization strategy [15,31].

The statistical analysis presented in Table 4 corroborates these findings, showing that the Pressure (P) factor was the primary driver of microbial inactivation (*p* < 0.001). According to the ANOVA results, while the Ripening stage (R) significantly influenced the initial microbial load of the fresh olive pomace, the magnitude of the HHP effect was sufficient to eliminate these biological differences. Specifically, the significant interaction observed between ripening stage and pressure (R × P) in Table 2 indicates that although olive pomaces from ripe olives presented a higher starting point of mesophilic bacteria, the treatment at 600 MPa achieved a uniform sanitization. This lack of variation in the final microbial status across different matrices (R × P interaction) highlights HHP as a robust and standardized preservation method for olive by-products [14,32].

Furthermore, the stabilization of mesophilic counts is critical to prevent the metabolic activity of spoilage organisms that could degrade the phenolic profile during storage [30]. It can be considered that HHP treatments ensure that olive pomace remains microbiologically safe without causing thermal degradations of bioactive compounds that are sensitive to heat treatment [8,33]. Therefore, HHP-stabilized pomace serves as a robust raw material for the development of enriched food products, meeting both the safety and nutritional demands of the current market [9,10].

Additionally, *Enterobacteriaceae* counts were below the detection limit (<1.0 log CFU g^−1^) in all analyzed samples, including the untreated raw pomace controls, which indicates a good initial hygienic quality of the raw material prior to any technological intervention.

### 3.3. Effects of HHP on Color and Chlorophyllic Pigments

The visual appearance of olive pomace is a primary quality attribute that influences consumer acceptance and reflects the integrity of its pigment fractions. The chromatic coordinates (L*, a*, b*) and chlorophyll content of the fresh and treated samples are summarized in Table 5, while the statistical significance of the factors and their interactions is presented in Table 2.

The ripening stage (R) was the most influential factor on the initial color and pigment concentration (*p* ≤ 0.001, Table 2). In both cultivars, the olive pomace samples from green ripening stage exhibited significantly higher L* and negative a* values (indicating greenness) compared to ripe samples. Conversely, advanced maturation shifted the coordinates toward lower L* and higher positive a* values, reflecting the chlorophyll degradation and the synthesis of secondary metabolites during fruit maturation, such as anthocyanins that give redness tones [17].

Regarding the impact of technological stabilization, HHP demonstrated an exceptional ability to preserve the macroscopic visual identity of the olive pomace. Contrary to the severe browning often associated with thermal processing, the application of pressure at 400 and 600 MPa did not induce drastic chromatic alterations. As detailed in Table 2, both the Pressure (P) factor and the Ripeness × Pressure (R × P) interaction were not significant (ns) for the total color difference ∆*E_HHP_*) in either cultivar. Consequently, the calculated ∆*E_HHP_* values (Table 5) remained largely below 3.0 across most batches—such as 2.13 and 2.69 for green *Morisca*—indicating that the pressure-induced color shifts are virtually imperceptible to the human eye [16]. These shifts were characterized by a decrease in L* and a slight loss of greenness (a*), likely associated with the initial steps of oxidative browning triggered by the release of endogenous enzymes during the pressure-induced cellular rupture [34,35].

Regarding chlorophyll pigments, the quantification of chlorophyll *a* and *b* revealed that treatment with HHP significantly increased the concentration of both chlorophylls in green olive pomace batches for the two cultivars. According to Table 5, chlorophyll *a* level in green *Morisca* pomace increased from 36.09 µg g^−1^ in the control to 42.40 µg g^−1^ after 600 MPa treatment. This increase, supported by the significant Ripening x Pressure interaction (*p* < 0.01, Table 2), is not due to pigment synthesis but to a “pressure-assisted extraction” effect. The mechanical stress of HHP disrupts the chloroplast membranes and the pectin-rich cell wall matrix, enhancing the extractability of pigments into the solvent during analysis [36,37].

Furthermore, our results align with previous studies [34,38], suggesting that HHP exerts a dual protective effect on the pigment degradation pathways. On one hand, it alters the activity of chlorophyllase, thereby preventing the rapid enzymatic cleavage of the phytol tail and the subsequent conversion of chlorophylls into chlorophyllides. On the other hand, because HHP is a non-thermal processing technology, it intrinsically avoids the heat-induced loss of the central magnesium ion that typically drives the degradation of chlorophylls into olive-brown pheophytins during standard thermal pasteurization. However, this protective effect was less evident in ripe olive pomaces, where the initial pigment load had already been reduced, and the R × P interaction (Table 2) suggests that the cultivar *‘Picual’* is more susceptible to chromatic degradation under high pressure when harvested at late stages. Consequently, for the production of green functional ingredients, HHP at 400–600 MPa serves as an effective tool to maximize pigment recovery, provided that the raw material is processed at an early ontogenetic stage.

### 3.4. Evolution of Total Phenolic Compounds Content and Total Antioxidant Activity

The functional value of olive pomace is intrinsically linked to its phenolic fraction, which dictates its overall antioxidant capacity and its potential as a health-promoting ingredient. The initial Total Phenolic Compounds Content (TPC) and Total Antioxidant Activity (TAA) of the untreated olive pomace (control samples, Table 6) were significantly influenced by the ripening stage (R) and cultivar (C) as shown in the ANOVA results (Table 2). As expected, green olive pomace samples exhibited significantly higher TPC and TAA values than ripe samples. This baseline difference reflects the endogenous enzymatic degradation of secoiridoids (such as oleuropein) into simpler, sometimes less antioxidative phenolic compounds during fruit ripening. Furthermore, the progression of maturation is accompanied by the evolution and synthesis of other phenolic classes, such as anthocyanins—which are responsible for the color shift towards reddish hues—implying the activation of distinct degradation mechanisms that deplete both complex and simple phenolic pools in the ripe fruit [8,17].

The impact of High Hydrostatic Pressure (HHP) on these bioactive fractions showed a matrix-dependent behavior. For TPC, the ANOVA (Table 2) indicated that the HHP treatment factor (P) was not significant (ns) for the *‘Morisca’* cultivar. As observed in Table 6, the TPC of *‘Morisca’* pomaces remained remarkably stable across all treatments, with values even showing a slight, though not statistically significant, increase after HHP application. Conversely, the HHP factor was highly significant (*p* ≤ 0.001) for the *‘Picual’* cultivar. However, the one-way ANOVA (Table 6) clarifies that the pressure-induced decrease in TPC for *‘Picual’* is not generalized; it is only statistically significant in the ripe batches, where it dropped from 5.48 to 4.44–4.46 mg GAE g^−1^, while the green *‘Picual’* batches maintained statistically equivalent TPC levels after pressurization.

The Total Antioxidant Activity (TAA) exhibited a different pattern, characterized by a higher sensitivity to pressure. In this case, HHP treatments induced a significant decrease in the TAA of green samples for both cultivars, dropping from 65.04 to 50.33–51.67 µmol TE g^−1^ in *‘Morisca’* and from 70.21 to 58.06–60.25 µmol TE g^−1^ in *‘Picual’* (Table 6). Ripe *‘Picual’* samples also suffered a significant reduction in their antioxidant capacity. However, as supported by the significant R × P interaction (*p* ≤ 0.01) in Table 2, the ripe *‘Morisca’* samples demonstrated a notable resilience, showing no significant loss of TAA following HHP treatment [9,10].

The observed decrease in phenolic content and antioxidant capacity at higher pressures can be attributed to the complex relationship between cell wall disruption and enzymatic stability. Although HHP is often reported to enhance the extractability of bioactive compounds by permeabilizing plant membranes [36,37], it can simultaneously trigger oxidative reactions if endogenous enzymes are not fully inactivated. In the olive matrix, polyphenol oxidase (PPO) and peroxidase (POX) show remarkable resistance to pressures up to 600 MPa, and their activities may even increase [30,35]. The mechanical stress of HHP likely induces the rupture of vacuolar structures, releasing sequestered phenols and facilitating their contact with residual active PPO. In the presence of residual oxygen within the vacuum-packed pomace, this contact promotes the oxidative polymerization of orthodiphenols, such as hydroxytyrosol, reducing the overall radical scavenging capacity even if the total quantifiable phenolic mass (TPC) remains relatively stable [16,39].

The relationship between the concentration of phenolic compounds and the radical scavenging capacity of the olive pomace was further evaluated through a Pearson correlation analysis. As illustrated in Figure 1, a strong positive linear correlation was observed between TPC and TAA across all samples studied. This relationship was found to be highly significant (R^2^ ≥ 0.92, *p* < 0.001), indicating that the phenolic fraction is the primary contributor to the antioxidant potential of the olive pomace, as has been previously documented in olive cake [39] and wet pomace [12]. These results are in agreement with previous findings in olive-derived matrices, where the presence of secoiridoids and their derivatives has been directly linked to the inhibition of oxidative processes [8,9]. The high correlation coefficient suggests that the TPC assay can serve as a robust and rapid indicator of the functional quality of HHP-stabilized ingredients. Furthermore, the persistence of this correlation after high-pressure processing confirms that, despite the mild oxidative shifts discussed previously, the antioxidant integrity of the pomace remains fundamentally dictated by its phenolic profile, supporting its application as a high-value functional additive in the food industry [7,10].

Finally, these results demonstrate that while HHP is highly effective for microbial stabilization, its application for the recovery of antioxidant ingredients must be carefully calibrated. The cultivar-specific response, particularly in the *‘Morisca’* cultivar, suggests that early-harvested raw materials are more suitable for technological stabilization, as they maintain a higher antioxidant potential. These findings provide a robust basis for the development of standardized and shelf-stable olive pomace ingredients with high nutritional value for the food industry based on HHP preservation, meeting the growing consumer demand for natural preservatives and functional additives [7,40].

### 3.5. Polyphenol Oxidase (PPO) Activity

The stability of endogenous enzymes, particularly polyphenol oxidase (PPO), is a critical factor in the non-thermal stabilization of olive pomace, as it directly influences the shelf-life and antioxidant integrity of the resulting ingredient. The residual PPO activity after HHP treatment is summarized in Table 6, with the statistical significance of the factors detailed in Table 2.

According to the ANOVA (Table 2), the ripening stage (R) exerted a highly significant influence (*p* ≤ 0.001) on the PPO activity for both the *Morisca* and *Picual* cultivars. Indeed, determination of PPO activity demonstrates that ripe pomaces possess a naturally higher PPO activity than green samples (Table 6). This physiological trend is particularly pronounced in the *Morisca* cultivar, where the enzymatic activity surged from approximately 0.03 Δ abs min^−1^ in green batches to over 0.18 Δ abs min^−1^ in ripe ones. This pronounced increase reflects the increased accessibility of the enzyme during the advanced stages of fruit ripening, a behavior consistent with previous observations in olive fruit maturation [16,17].

Conversely, the application of High Hydrostatic Pressure (HHP) at 400 and 600 MPa failed to induce any enzymatic inactivation. The ANOVA (Table 2) confirms that both the Pressure (P) factor and the interaction between ripeness and pressure (R × P) were not significant (ns) for either cultivar. Consequently, as shown in Table 6, the PPO activity of the HHP-treated matrices remained statistically equivalent to their respective untreated controls. This profound baroresistance indicates that pressures up to 600 MPa are entirely insufficient to induce the irreversible structural denaturation required to deactivate the olive PPO. The enzyme’s globular structure and strong stabilization within the membrane-bound fraction of the olive matrix often require pressures exceeding 800 MPa or combinations with thermal treatment for total denaturation [30,39].

The non-significant R × P interaction indicates that the persistence of PPO is completely uniform across all batches. However, since ripe pomaces possess a substantially higher baseline of active PPO, the pressure-induced cell package disruption releases vacuolar phenolic substrates, triggering a more intense oxidative cascade in mature tissues than in green ones. This enzymatic persistence, combined with the pressure-induced release of phenolic substrates from the vacuoles, explains the significant losses in hydroxytyrosol and oleuropein observed in the HPLC profile [34,35].

### 3.6. Effects of HHP on the Individual Phenolic Compound Profile

To further elucidate the mechanisms driving the changes in the antioxidant capacity of the *olive pomace*, the individual phenolic profile was characterized via HPLC-DAD-FLD. The quantification of major phenolic alcohols, secoiridoids and minor constituents is detailed in Table 7. The statistical significance of the experimental factors and their interactions is detailed in the two-way ANOVA results in Table 3. The baseline composition of the untreated samples was dominated by hydroxytyrosol, tyrosol, and oleuropein, which are widely recognized as the primary bioactive markers of olive-derived matrices [7,17,25]. Consistent with the TPC trends, the initial concentrations of these individual compounds were highly dependent on the ripening stage (Table 3; *p* ≤ 0.05). Green pomaces of both cultivars exhibited higher levels of oleuropein (Table 7), which naturally undergoes enzymatic hydrolysis during fruit maturation, leading to a relative increase in free hydroxytyrosol in the ripe control batches [17].

The application of HHP induced selective shifts in the phenolic fingerprint of the pomace. For hydroxytyrosol, a downward trend after pressure treatment was observed across all four pomace types; however, the two-way ANOVA (Table 3) revealed that the HHP treatment factor was non-significant (ns) for the *Morisca* cultivar, whereas it was highly significant (*p* ≤ 0.001) for *Picual*. The one-way ANOVA (Table 7) clarifies that this pressure-induced reduction is uniquely significant in the green *Picual* batch, where values dropped from 85.04 to 68.43 µg mL^−1^ extract, while the other matrices maintained statistically equivalent levels to their controls. This distinct behavior demonstrates that hydroxytyrosol retention is highly dependent on both the specific cultivar matrix and the applied technological treatment. This varietal divergence can be fundamentally attributed to differences in the endogenous enzymatic equipment. The specific isoforms and expressions of enzymes involved in oleuropein transformation vary widely among Spanish olive cultivars [41]. Furthermore, genome-wide analyses reveal that the polyphenol oxidase (PPO) gene family in *Olea europaea* possesses distinct transcriptional profiles and structural characteristics among cultivars, directly conditioning the specific rate of enzymatic browning and substrate affinity under stressful processing environments [42].

This selective degradation strongly supports the hypothesis of pressure-induced enzymatic oxidation discussed in Section 3.3. Both hydroxytyrosol and oleuropein possess an *ortho*-diphenolic structure, making them the preferred substrates for polyphenol oxidase (PPO) [16]. The mechanical disruption of the cellular compartmentalization at 600 MPa facilitates the rapid interaction between these substrates and the residual active PPO, leading to their conversion into highly reactive quinones and subsequent polymerization [35].

In contrast, monophenols like tyrosol exhibit a different stability pattern. Lacking the adjacent hydroxyl groups required for rapid catecholase activity, monophenols are chemically less susceptible to direct PPO-mediated oxidation, demonstrating a naturally superior resilience to pressure-induced enzymatic breakdown compared to their ortho-diphenolic counterparts [30]. However, it must be noted that in the *Picual* cultivar, tyrosol levels were still significantly reduced by HHP processing (*p* ≤ 0.001, Table 3), further emphasizing the intense oxidative environment triggered in this specific matrix.

Interestingly, the concentration of tyrosol—a monophenol—exhibited greater stability under high-pressure conditions compared to its *ortho*-diphenolic counterparts. Since tyrosol lacks the 1,2-dihydroxybenzene moiety, it is significantly less susceptible to direct PPO-mediated oxidation [30]. This differential behavior between tyrosol and hydroxytyrosol provides robust chemical evidence that the overall loss of antioxidant activity is not due to physical degradation caused by the pressure itself, but rather to the cascade of enzymatic oxidative reactions triggered by the compromised integrity of the cellular matrix.

Minor phenolic constituents were evaluated to assess their technological stability against pressure-induced enzymatic degradation (Table 7). The verbascoside + rutin fraction displayed divergent behaviors between the two cultivars; while it remained statistically unaltered in *Morisca* pomace (ns, Table 3), it exhibited a highly significant decrease following HHP treatment in *Picual* (*p* ≤ 0.001), where the mature control fell drastically from 7.83 to 3.11 µg mL^−1^ extract at 600 MPa [27,35]. In contrast, p-coumaric acid and apigenin-7-O-glucoside demonstrated remarkable resilience, or even an increasing trend, such as green *Morisca*. As monophenols, they lack the catechol structure necessary for rapid enzymatic browning, and their stability is further supported by the pressure-assisted extraction effect from the cell wall matrix [30,36]. Furthermore, luteolin-7-O-glucoside showed higher stability than simple phenolic alcohols, suggesting that its glycosylation and complex flavone structure confer protection against oxidative enzyme cascades, showing no significant degradation under pressure (ns, Table 3) [7,9].

Despite these oxidative shifts, the HHP-treated pomaces retained a substantial concentration of high-value secoiridoids, especially when processed at early ripening stages (*Morisca* green). Considering that traditional thermal pasteurization causes severe, non-selective thermal degradation of these thermolabile compounds, HHP remains an excellent alternative for bio-stabilization [8]. HHP-treated *orujo* still retains physiologically relevant doses of main phenols alongside highly stable minor fractions and flavone glycosides [9,43].

### 3.7. Effect of HHP on the Fatty Acid Profile

Although mechanical extraction removes most of the oil during the milling process, the residual lipid fraction in olive pomace remains a highly valuable nutritional source. The detailed composition of individual fatty acid methyl esters (FAMEs) for the untreated and HHP-treated pomace samples is presented in Table 8, while the statistical significance of the main factors and their interactions is detailed in the ANOVA results in Table 2.

The baseline lipid profile was predominantly composed of oleic acid (C18:1), followed by palmitic acid (C16:0) and linoleic acid (C18:2). The initial distribution of these major fatty acids was highly dependent on the cultivar [17]. The *Picual* cultivar inherently exhibited a much higher relative percentage of oleic acid (exceeding 74% across all batches) compared to the *Morisca* cultivar (ranging from 60.4% to 68.2%). Conversely, the *Morisca* pomace was characterized by a notably higher proportion of linoleic acid (11.7% to 19.0%) compared to *Picual* (6.6% to 7.9%).

The ripening stage (R) emerged as the primary driver of changes in the lipid profile, showing high statistical significance (*p* ≤ 0.001) for most fatty acids in both cultivars according to the two-way ANOVA (Table 2). As observed in Table 8, advanced ripeness in the *Morisca* pomace led to a significant decrease in oleic acid (from 68.21% in the green control to 60.41% in the ripe control) and a proportional increase in linoleic acid (from 11.74% to 18.47%). In contrast, the *Picual* cultivar exhibited a much more stable profile during ripening, maintaining its oleic acid levels practically unaltered during ripening (with no significant differences indicated in the ANOVA for C18:1 in *Picual*, Table 8).

Regarding the impact of technological stabilization, HHP demonstrated adequate stabilization of the valuable unsaturated fatty acids. The two-way ANOVA (Table 2) revealed that both the Pressure (P) factor and the Ripeness × HHP interaction were not significant (*ns*) for oleic (C18:1) and linoleic (C18:2) acids in either cultivar. This structural stability is corroborated by the one-way ANOVA in Table 8: within each ripeness stage, the relative percentages of these unsaturated fatty acids in the 400 and 600 MPa treated samples shared the same statistical significance as their respective untreated controls.

However, HHP did produce a slight but significant effect on certain saturated fatty acids, particularly palmitic acid (C16:0). The two-way ANOVA (Table 2) showed a highly significant pressure effect (*p* ≤ 0.001) for C16:0 in both cultivars, along with a significant Ripeness x HHP interaction (*p* ≤ 0.05) in the *Picual* pomace. As detailed in Table 8, treatments at 400 and 600 MPa induced slight reductions in palmitic acid compared to the control (dropping from 14.75% to 14.33% in green *Morisca*).

Despite these minor statistical shifts in the saturated fraction, in contrast to the phenolic fraction, the overall core lipid matrix exhibited a remarkable stability under high pressure. The stability of the major unsaturated fatty acids under high-pressure conditions is governed by thermodynamic principles. Unlike conventional thermal pasteurization, which can supply sufficient activation energy to trigger the auto-oxidation of double bonds or lipid isomerization, HHP up to 600 MPa primarily affects non-covalent interactions [44]. The applied pressure lacks the thermodynamic energy required to disrupt the covalent bonds of the hydrocarbon chains in oleic and linoleic acids [8,44]. Consequently, HHP enables the effective microbial sanitation of the pomace without inducing lipid rancidity, thereby preserving the nutritional integrity and sensory viability of the pomace as a high-quality functional ingredient.

### 3.8. Principal Components Analysis (PCA)

To evaluate how experimental factors define the overall quality fingerprint of the olive pomace, a Principal Component Analysis (PCA) was performed. The first model (Figure 2a–c), based exclusively on physicochemical, chromatic and individual nutritional parameters (87.2% total variance), revealed a clustering driven primarily by ontogenetic stage and genetic origin (Figure 2a–c).

PC1 (56.5%) effectively discriminated samples by ripeness, with green pomaces correlating with higher total phenols, chlorophylls, and oleuropein [17,27] while ripe samples shifted toward higher polyphenol oxidase (PPO) activity and redness (a*) [16]. Meanwhile, PC2 (30.7%) separated the cultivars, positioning the *Picual* cultivar with its characteristic high oleic acid (C18:1) content opposite to the linoleic acid-rich *Morisca* pomace. Crucially, when grouped by HHP treatment (Figure 2c), HHP-treated samples closely overlapped with their untreated controls, visually confirming that high-pressure processing preserves the intrinsic macro-physicochemical and nutritional identity of the raw material [33,36].

To assess the true technological efficacy of the stabilization process, a second PCA model (Figure 2d–f) integrated both microbiological and physicochemical parameters (82.7% total variance) (Figure 2d–f). While the segregation by ripeness remained robust along PC1, the inclusion of microbial data restructured the sample distribution along PC2, overlapping the cultivars in the case of the ripe pomaces. The score plot grouped by treatment (Figure 2f) demonstrated a discrimination between the untreated controls and the HHP-stabilized samples. The loading plot (Figure 2d) confirmed that this treatment separation was driven by the microbial load, pulling the contaminated untreated samples toward the positive side of PC2, while all HHP-treated matrices (400 and 600 MPa) clustered tightly on the negative side due to the complete inactivation of molds, yeasts, and mesophilic bacteria [30,31].

Ultimately, this multivariate approach effectively encapsulates the dual benefit of the proposed stabilization strategy. The PCA models provide a robust visual and statistical proof that HHP treatments successfully guarantee the microbiological safety of the pomace (as visualized in Model 2) while simultaneously safeguarding its natural, high-value physicochemical and lipid profile (as visualized in Model 1). This dual capability confirms that the targeted application of high-pressure processing, particularly on early-harvested pomaces, enables the effective upcycling of olive pomace into stable, functionally rich ingredients for the food industry, avoiding the severe compositional degradation typically associated with conventional thermal pasteurization [8,9].

## 4. Conclusions

The application of High Hydrostatic Pressure (HHP) at 400 and 600 MPa represents a highly effective, non-thermal strategy for the bio-stabilization of olive pomace. HHP guarantees microbiological sanitation by reducing aerobic mesophiles, molds, and yeasts below analytical detection limits across different cultivars and ripening stages across both *Morisca* and *Picual* cultivars. Concurrently, this technology exceptionally preserves the macroscopic visual appearance of the matrix, yielding total color differences (ΔE_HHP_) below the human perception threshold. Furthermore, this stability preserves its lipid profile in particular, safeguarding the high-value unsaturated fatty acids, such as oleic and linoleic acids, from oxidative rancidity.

However, the technological success of HHP involves a selective, matrix-dependent biochemical trade-off driven by the baroresistance of endogenous polyphenol oxidase (PPO), which remains active post-treatment. While pressure-induced microstructural disruption successfully enhances the extractability of valuable pigments like chlorophylls in green batches, it simultaneously releases phenolic compounds from vacuolar structures. This cellular de-compartmentalization triggers a selective oxidative cascade where *ortho*-diphenols (such as hydroxytyrosol, oleuropein, and the verbascoside+rutin fraction) decrease due to active PPO catecholase activity, inducing a significant reduction in total antioxidant activity (TAA), particularly in green pomaces and ripe *Picual* samples. Conversely, monophenols (tyrosol, *p*-coumaric acid) and glycosylated flavones remain highly stable throughout the processing due to absence of the catechol moiety in their chemical structure. Crucially, despite these localized oxidative shifts, a strong positive linear correlation (R^2^ ≥ 0.92, *p* < 0.001) between total phenolic content (TPC) and TAA is strictly maintained across all matrices. This statistical persistence proves that the radical scavenging capacity of the stabilized *orujo* remains fundamentally governed by its phenolic profile, confirming that the overall functional integrity of the by-product is preserved after high-pressure processing.

In conclusion, utilizing HHP-stabilized olive pomace as a functional food additive requires a strategic compromise between achieving microbial safety and managing the selective retention of its phytochemical profile. Processing olive pomace at early maturity stages (green pomaces) represents a suitable approach, as these matrices possess a significantly lower baseline of active PPO and a more resilient antioxidant pool. However, the industrial scalability of this approach will ultimately depend on future cost–benefit analyses, given the high-volume and low-unit-value nature of the commodity. Furthermore, because HHP does not inactivate PPO, a complementary mild stabilization step (such as vacuum drying, mild blanching, or the addition of natural antioxidants) might be necessary to fully halt enzymatic browning during the long-term storage of the ingredient. Overall, when properly integrated into the processing pipeline, HHP serves as a highly valuable and non-thermal upcycling tool to deliver microbiologically safe and lipid-stable functional formulations.

## Figures and Tables

**Figure 1:**
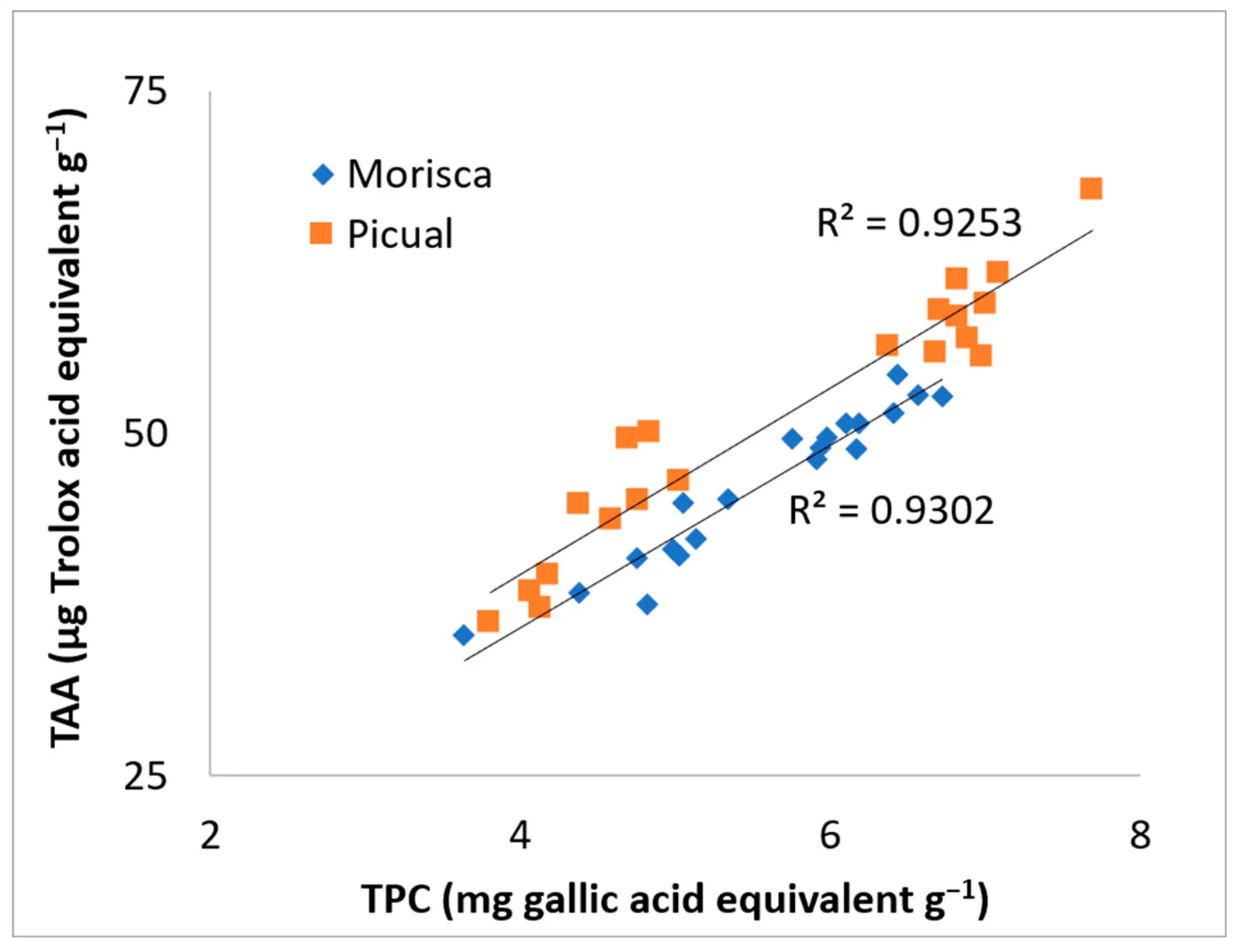
Correlation between total antioxidant capacity (TAA) and total phenolic compounds content (PC) for *‘Morisca’* and *‘Picual’* pomaces. R^2^: Pearson’s correlation (significance ≤ 0.001).

**Figure 2:**
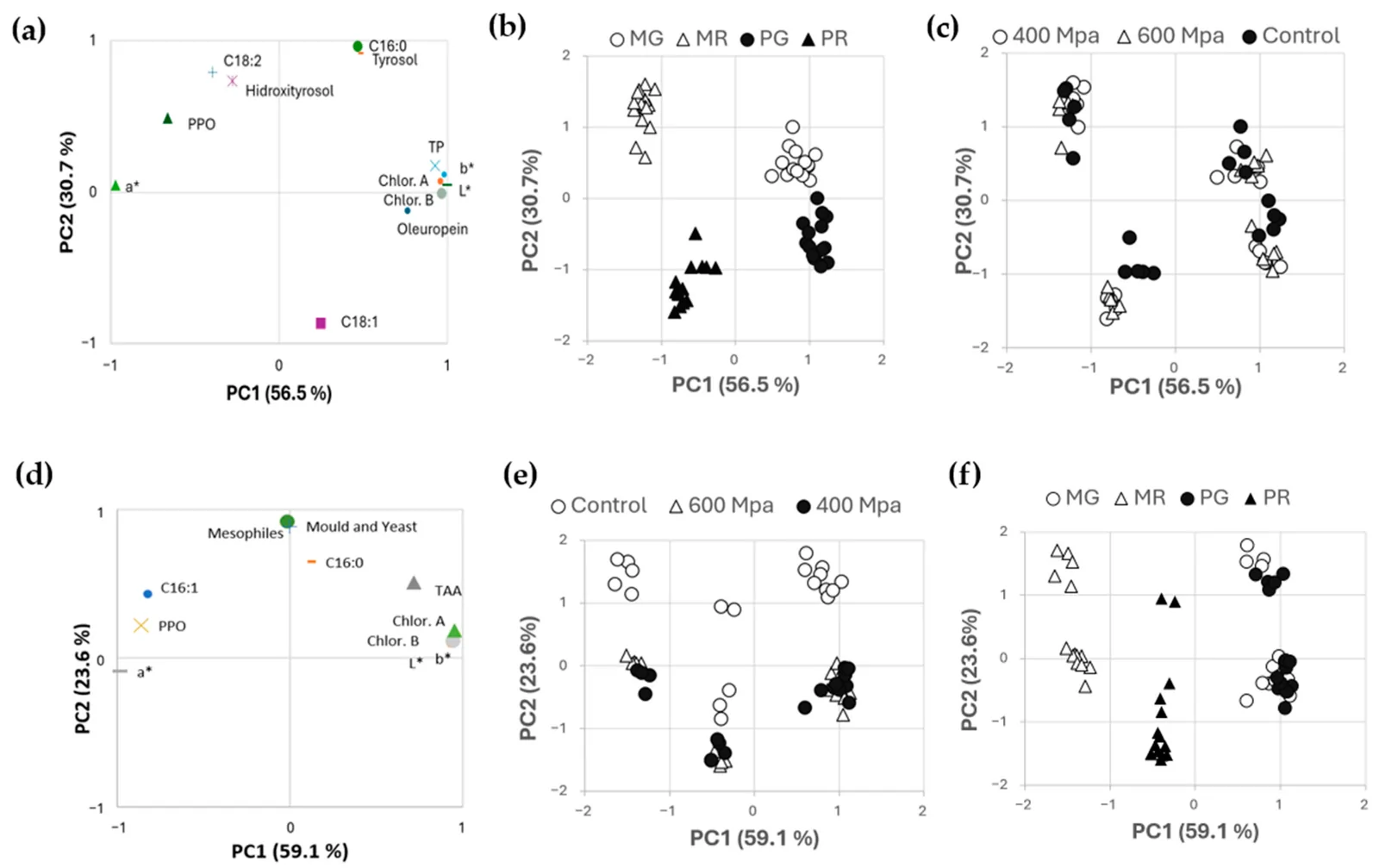
Biplot of the loadings and scores of the two-dimensional PCA models. (**a**–**c**) correspond to the modeling based on the physicochemical parameters. (**d**–**f**) correspond to modeling based on the microbiological and physicochemical parameters. MG: *Morisca* green. MR: *Morisca* ripe. PG: *Picual* green. PR: *Picual* ripe.

**Table 1:** Characterization of the olive fruits.

	*Morisca*		*Picual*	
	Green	Ripe	Green	Ripe
RI (Ripening index, range 0–7)	0.30 ± 0.30	4.96 ± 0.10	0.35 ± 0.20	3.12 ± 0.70
Sanitary state (% healthy fruits)	98.2 ± 2.4	76.2 ± 11.2	100.0 ± 0.1	87.9 ± 8.0
Weight of 100 fruits (g)	503.5 ± 91.3	693.0 ± 80.4	239.5 ± 91.8	471.9 ± 173.7
Moisture content (%)	56.3 ± 3.9	57.6 ± 3.1	51.4 ± 3.8	50.8 ± 2.9
Fat content on a dry basis (%)	40.7 ± 2.7	52.7 ± 2.3	33.3 ± 5.2	44.5 ± 6.3
Fat content on wet basis (%)	17.8 ± 2.3	22.3 ± 2.1	16.2 ± 3.2	21.7 ± 1.9

**Table 2:** Analysis of variance (F-values) showing the effect of ripeness, High Hydrostatic Pressure (HHP), and their interaction on the physicochemical and microbiological properties of *‘Morisca’* and *‘Picual’* olive pomaces.

	*Morisca*			*Picual*		
	Ripeness	HHP	Ripeness × HHP	Ripeness	HHP	Ripeness × HHP
Mesophiles	0.20 ^ns^	111.51 ***	4.54 *	5.57 *	18.57 ***	0.34 ^ns^
Molds and yeasts	1.70 ^ns^	371.06 ***	0.32 ^ns^	10.96 **	62.45 ***	2.74 ^ns^
L*	1140.72 ***	1.54 ^ns^	2.60 ^ns^	261.25 ***	11.96 ***	1.44 ^ns^
a*	14,189.72 ***	11.77 ***	6.09 **	5415.91 ***	2.41 ^ns^	38.34 ***
b*	2253.19 ***	15.46 ***	4.42 *	756.90 ***	4.12 *	0.37 ^ns^
ΔE_HHP_	11.24 **	0.01 ^ns^	0.04 ^ns^	1.94 ^ns^	0.02 ^ns^	0.02 ^ns^
TPC	143.02 ***	0.01 ^ns^	2.43 ^ns^	210.02 ***	8.68 ***	3.03 ^ns^
TAA	89.19 ***	23.85 ***	5.87 **	120.21 ***	18.38 ***	0.50 ^ns^
PPO	211.94 ***	2.10 ^ns^	1.67 ^ns^	44.22 ***	0.40 ^ns^	1.29 ^ns^
Chlorophyll a	4152.58 ***	13.50 ***	19.19 ***	459.49 ***	5.40 *	9.06 **
Chlorophyll b	1259.21 ***	6.69 **	11.07 ***	246.92 ***	4.01 *	5.75 **
Lauric acid (C12:0)	94.04 ***	0.63 ^ns^	2.10 ^ns^	57.94 ***	1.98 ^ns^	24.58 ***
Mirstic acid (C14:0)	103.18 ***	5.55 *	0.25 ^ns^	34.80 ***	9.51 ***	5.09 *
Palmitic acid (C16:0)	78.09 ***	22.66 ***	0.32 ^ns^	2336.23 ***	59.31 ***	4.92 *
Palmitoleic acid (C16:1)	2190.35 ***	65.78 ***	6.81 **	5.63 *	56.21 ***	1.48 ^ns^
Margaric acid (C17:0)	3.31 ^ns^	4.90 *	0.26 ^ns^	0.30 ^ns^	0.96 ^ns^	1.26 ^ns^
Margaroleic acid (C17:1)	16.28 ***	1.87 ^ns^	2.62 ^ns^	58.94 ***	7.04 **	7.31 **
Estearic acid (C18:0)	2582.64 ***	7.68 **	0.34 ^ns^	1042.02 ***	17.02 ***	6.16 **
cis-9-oleic acid (C18:1)	258.76 ***	0.82 ^ns^	0.59 ^ns^	7.02 *	0.72 ^ns^	1.07 ^ns^
Linoleic acid (C18:2. cis.cis)	1333.72 ***	2.90 ^ns^	0.26 ^ns^	22.01 ***	0.77 ^ns^	1.18 ^ns^
Linolenic acid (C18:3)	79.19 ***	1.58 ^ns^	3.91 *	349.84 ***	2.79 ^ns^	3.03 ^ns^
Araquic acid (C20:0)	11.82 **	3.25 ^ns^	1.28 ^ns^	0.98 ^ns^	14.00 ***	0.41 ^ns^
Gadoleic acid (C20:1)	140.16 ***	28.04 ***	34.83 ***	48.68 ***	10.80 ***	8.12 **

TPC: total phenolic compounds content; TAA: total antioxidant activity; PPO: polyphenol oxidase. Values represent the F-ratio from the two-way ANOVA. Significance levels: ns (not significant, *p* > 0.05); * *p* ≤ 0.05; ** *p* ≤ 0.01; *** *p* ≤ 0.001. Degrees of freedom (df) for Ripeness = 1, HHP = 2, Ripeness × HHP = 2, Error = 24.

**Table 3:** Interaction between olive ripeness and HHP treatment factors in the individual phenolic compounds profile of *‘Morisca’* and *‘Picual’* olive pomaces.

	*Morisca*			*Picual*		
	Ripeness	HHP	Ripeness × HHP	Ripeness	HHP	Ripeness × HHP
Apigenin-7-O-glucoside	0.25 ^ns^	5.24 *	0.38 ^ns^	31.07 ***	0.11 ^ns^	0.50 ^ns^
Hydroxytyrosol	24.79 ***	1.04 ^ns^	0.46 ^ns^	1.422 ^ns^	23.728 ***	2.123 ^ns^
Luteolin-7-O-glucoside	3.54 ^ns^	4.38 *	1.14 ^ns^	23.45 ***	2.62 ^ns^	0.35 ^ns^
Oleuropein	74.80 ***	3.41 *	2.43 ^ns^	224.57 ***	9.73 ***	3.60 *
p-Coumaric acid	421.91 ***	7.39 **	2.90 ^ns^	332.82 ***	5.75 **	1.98 ^ns^
Tyrosol	7.06 *	0.78 ^ns^	12.47 ***	134.13 ***	33.64 ***	2.75 ^ns^
Verbascoside + runtin	4.98 *	1.24 ^ns^	0.66 ^ns^	38.49 ***	40.85 ***	3.58 *
% Hidrotyrosol	96.83 ***	1.96 ^ns^	0.71 ^ns^	195.18 ***	3.08 ^ns^	3.19 ^ns^

Sig.: significance. ANOVA test significance levels: ns *p* > 0.05; * *p* ≤ 0.05; ** *p* ≤ 0.01; *** *p* ≤ 0.001.

**Table 4:** Microbiological loads (log CFU g^−1^) of *‘Morisca’* and *‘Picual’* olive pomaces with different HHP treatments and two ripeness stages.

	Green			Ripe			
	No HHP	400 MPa	600 MPa	No HHP	400 MPa	600 MPa	Sig.
*‘Morisca’ cv.*							
Mesophiles	3.20 ± 0.11 a	1.41 ± 0.27 b	1.41 ± 0.40 b	2.85 ± 0.23 a	1.56 ± 0.31 b	1.73 ± 0.12 b	***
Molds and yeasts	3.17 ± 0.13 a	0.18 ± 0.40 c	0.95 ± 0.12 b	3.00 ± 0.43 a	nd c	0.94 ± 0.08 b	***
*‘Picual’ cv.*							
Mesophiles	2.77 ± 0.27 a	1.75 ± 0.14 bc	1.76 ± 0.13 bc	2.46 ± 1.02 ab	1.46 ± 0.26 c	1.16 ± 0.22 c	***
Molds and yeasts	3.06 ± 0.35 a	0.99 ± 0.21 b	nd b	2.08 ± 1.27 a	nd b	nd b	***

Total mesophiles (log CFU g^−1^). Total molds and yeasts (log CFU g^−1^). nd: no detect. Detection limit below 1.0 log CFU g^−1^. Mean values followed by different letters indicate the existence of significant differences between samples by the Tukey test (*p* ≤ 0.05). Sig.: Significance. ANOVA test significance levels: *** *p* ≤ 0.001.

**Table 5:** Instrumental color parameters of *‘Morisca’* and *‘Picual’* olive pomaces with different HHP treatments and two ripening stages.

	Green			Ripe			
	No HHP	400 MPa	600 MPa	No HHP	400 MPa	600 MPa	Sig.
*‘Morisca’ cv.*							
L*	43.01 ± 0.87 a	44.07 ± 1.34 a	44.35 ± 0.73 a	34.07 ± 0.51 b	33.91 ± 0.57 b	33.91 ± 0.55 b	***
a*	−1.11 ± 0.18 c	−1.10 ± 0.40 c	−1.24 ± 0.20 c	8.45 ± 0.12 a	7.68 ± 0.13 b	8.03 ± 0.16 b	***
b*	20.18 ± 0.84 b	21.80 ± 1.10 a	22.47 ± 0.65 a	9.95 ± 0.37 c	10.57 ± 0.15 c	10.75 ± 0.25 c	***
Chroma	20.21 ± 0.84 b	21.83 ± 1.11 a	22.50 ± 0.66 a	13.02 ± 0.40 c	13.07 ± 0.15 c	13.41 ± 0.30 c	***
Hue	93.13 ± 0.38 a	92.86 ± 0.95 a	93.15 ± 0.42 a	49.53 ± 0.78 c	54.00 ± 0.58 b	53.24 ± 0.24 b	***
ΔE__HHP_	-	2.13±1.46	2.69±0.90	-	1.12 ± 0.23	1.06 ± 0.20	ns
Chlorophyll a	36.09 ± 1.74 b	43.73 ± 3.02 a	42.40 ± 1.73 a	3.35 ± 0.27 c	2.53 ± 0.23 c	3.07 ± 0.43 c	***
Chlorophyll b	14.14 ± 0.62 b	17.81 ± 2.00 a	17.00 ± 1.32 a	2.70 ± 0.34 c	1.91 ± 0.31 c	2.97 ± 0.67 c	***
*‘Picual’ cv.*							
L*	44.48 ± 0.92 a	43.07 ± 1.53 a	43.17 ± 1.47 a	38.99 ± 0.44 b	36.31 ± 0.86 c	36.12 ± 0.93 c	***
a*	−1.93 ± 0.22 d	−1.23 ± 0.37 c	−1.36 ± 0.35 c	5.55 ± 0.11 a	4.61 ± 0.10 b	4.52 ± 0.10 b	***
b*	22.81 ± 0.82 a	21.43 ± 1.16 a	21.63 ± 1.54 a	13.40 ± 0.40 b	12.67 ± 0.37 b	12.73 ± 0.38 b	***
Chroma	22.89 ± 0.83 a	21.46 ± 1.17 a	21.67 ± 1.56 a	14.13 ± 0.59 b	13.49 ± 0.33 b	13.51 ± 0.38 b	***
Hue	94.82 ± 0.44 a	93.26 ± 0.85 a	93.56 ± 0.71 a	63.93 ± 7.73 c	69.99 ± 0.81 bc	70.46 ± 0.53 b	***
ΔE__HHP_	-	2.17 ± 1.85	2.15 ± 1.79	-	2.96 ± 0.82	3.13 ± 0.94	ns
Chlorophyll a	30.66 ± 1.99 b	43.01 ± 5.46 a	41.06 ± 6.85 a	8.97 ± 1.02 c	7.35 ± 0.47 c	7.35 ± 0.47 c	***
Chlorophyll b	12.81 ± 1.22 b	17.87 ± 2.61 a	17.46 ± 3.39 a	4.89 ± 1.02 c	4.19 ± 0.52 c	4.19 ± 0.52 c	***

Chlorophyll a (μg g^−1^), chlorophyll b (μg g^−1^). Mean values followed by different letters indicate the existence of significant differences between samples by the Tukey test (*p* ≤ 0.05). Sig.: Significance. ANOVA test significance levels: ns *p* > 0.05; *** *p* ≤ 0.001.

**Table 6:** Contents of total polyphenols. Total hydrophilic antioxidant activity (AAH). Polyphenol oxidase activity (PPO), and chlorophyll contents in *Morisca* and *Picual* olive pomaces with different HHP treatments and two ripening states (Mean values ± standard deviation).

	Green			Ripe			
	No HHP	400 MPa	600 MPa	No HHP	400 MPa	600 MPa	Sig.
*‘Morisca’ cv.*							
TPC	6.34 ± 0.33 a	6.07 ± 0.25 a	6.37 ± 0.30 a	4.82 ± 0.35 b	5.07 ± 0.22 b	4.80 ± 0.31 b	***
TAA	65.04 ± 5.09 a	50.33 ± 0.99 b	51.67 ± 2.33 b	46.72 ± 4.11 bc	42.92 ± 3.94 bc	40.18 ± 3.59 c	***
PPO	0.03 ± 0.01 b	0.02 ± 0.01 b	0.03 ± 0.01 b	0.18 ± 0.04 a	0.16 ± 0.04 a	0.21 ± 0.04 a	***
*‘Picual’ cv.*							
TPC	7.21 ± 0.61 a	7.07 ± 0.38 a	6.75 ± 0.23 a	5.48 ± 0.36 b	4.44 ± 0.50 c	4.46 ± 0.32 c	***
TAA	70.21 ± 3.19 a	60.25 ± 4.74 b	58.06 ± 2.27 bc	51.75 ± 3.55 c	42.91 ± 6.00 d	43.26 ± 4.47 d	***
PPO	0.02 ± 0.00 bc	0.01 ± 0.01 c	0.01 ± 0.01 c	0.04 ± 0.01 ab	0.04 ± 0.01 ab	0.04 ± 0.01 a	***

TPC: total polyphenolic compounds content (mg gallic acid equivalent g^−1^). TAA: total antioxidant activity (μg Trolox acid equivalent g^−1^). PPO: polyphenol oxidase (Δ absorbance minute^−1^). Means values followed by different letters indicate the existence of significant differences between samples by the Tukey test (*p* ≤ 0.05). Sig.: significance. ANOVA test significance levels: *** *p* ≤ 0.001.

**Table 7:** Effect of the ripening stage and HHP treatment on individual phenolic profile for *Morisca* and *Picual* olive pomaces (μg ml^−1^ extract; mean value ± standard deviation).

	Green			Ripe			
	Control	400 MPa	600 MPa	Control	400 MPa	600 MPa	Sig.
*‘Morisca’ cv.*							
Hydroxytyrosol	94.67 ± 9.07 abc	83.50 ± 4.66 c	87.22 ± 4.98 bc	111.01 ± 14.11 a	109.25 ± 13.97 a	106.38 ± 15.29 ab	**
Oleuropein	6.33 ± 1.47 a	5.49 ± 0.41 ab	7.32 ± 1.87 a	3.60 ± 1.63 cb	1.78 ± 0.12 c	2.19 ± 0.69 c	***
Verbascoside + rutin	6.80 ± 0.96	5.74 ± 0.62	6.80 ± 1.00	7.91 ± 1.77	7.27 ± 0.93	7.11 ± 1.59	ns
Tyrosol	5.80 ± 0.59 a	4.64 ± 0.10 bc	4.98 ± 0.26 abc	4.02 ± 0.86 c	5.36 ± 0.45 ab	4.41 ± 0.73 bc	***
Luteolin-7-O-glucoside	3.37 ± 0.59	3.33 ± 0.84	3.78 ± 0.24	2.43 ± 0.28	2.72 ± 0.57	3.82 ± 1.32	ns
Apigenin-7-O-glucoside	0.58 ± 0.58	0.88 ± 0.38	1.11 ± 0.26	0.33 ± 0.74	0.97 ± 0.25	1.03 ± 0.09	ns
p-Coumaric acid	2.34 ± 0.17 b	2.46 ± 0.27 ab	2.73 ± 0.15 a	1.01 ± 0.18 c	1.32 ± 0.09 c	1.21 ± 0.15 c	***
*‘Picual’ cv.*							
Hydroxytyrosol	85.04 ± 9.30 ab	68.43 ± 4.04 c	70.64 ± 5.36 bc	97.51 ± 6.88 a	66.74 ± 11.02 c	70.77 ± 10.90 bc	***
Oleuropein	15.52 ± 1.58 a	11.10 ± 3.09 b	15.38 ± 1.81 a	5.85 ± 2.16 c	3.08 ± 0.22 c	3.01 ± 0.25 c	***
Verbascoside + rutin	9.54 ± 2.39 a	5.17 ± 0.77 bc	7.34 ± 0.75 ba	7.83 ± 0.87 a	3.22 ± 0.51 c	3.11 ± 0.49 c	***
Tyrosol	4.91 ± 0.36 a	3.38 ± 0.15 b	3.60 ± 0.19 b	3.01 ± 0.72 b	2.21 ± 0.18 c	2.2 ± 0.10 c	***
Luteolin-7-O-glucoside	4.51 ± 0.80 a	3.58 ± 1.07 abc	4.50 ± 0.34 ab	3.00 ± 0.93 abc	2.56 ± 0.71 c	2.97 ± 0.49 bc	**
Apigenin-7-O-glucoside	0.49 ± 0.45 c	0.72 ± 0.34 ab	0.52 ± 0.04 c	1.41 ± 0.8 ab	1.35 ± 0.17 b	1.49 ± 0.14 b	**
p-Coumaric acid	2.09 ± 0.33 ab	1.74 ± 0.25 b	2.12 ± 0.17 a	0.82 ± 0.10 c	0.62 ± 0.07 c	0.67 ± 0.078 c	***

Values expressed as µg mL^−1^. Mean values followed by different letters indicate the existence of significant differences between samples by the Tukey test (*p* ≤ 0.05). Sig.: significance. ANOVA test significance levels: ns *p* > 0.05; *p* ** ≤ 0.01. *** *p* ≤ 0.001.

**Table 8:** Fatty acid profile of *Morisca* and *Picual* olive pomaces with different HHP treatments and two maturity states.

	Green			Ripe			
	No HHP	400 MPa	600 MPa	No HHP	400 MPa	600 MPa	Sig.
*‘Morisca’ cv.*							
Lauric (C12:0)	0.07 ± 0.01 a	0.08 ± 0.01 a	0.07 ± 0.01 a	0.05 ± 0.00 b	0.05 ± 0.00 b	0.05 ± 0.00 b	***
Miristic (C14:0)	0.08 ± 0.01 a	0.08 ± 0.01 a	0.07 ± 0.01 a	0.05 ± 0.00 b	0.05 ± 0.00 b	0.04 ± 0.00 b	***
Palmitic (C16:0)	14.75 ± 0.34 a	14.38 ± 0.18 b	14.33 ± 0.11 b	14.26 ± 0.04 b	13.82 ± 0.08 c	13.72 ± 0.08 c	***
Palmitoleic (C16:1)	1.05 ± 0.05 c	0.97 ± 0.02 d	0.98 ± 0.01 d	1.53 ± 0.01 a	1.39 ± 0.02 b	1.37 ± 0.01 b	***
Margaric (C17:0)	0.05 ± 0.00	0.05 ± 0.00	0.05 ± 0.00	0.05 ± 0.00	0.05 ± 0.00	0.05 ± 0.00	ns
Margaroleic (C17:1)	0.06 ± 0.01 b	0.06 ± 0.00 ab	0.07 ± 0.00 ab	0.07 ± 0.00 a	0.07 ± 0.00 a	0.07 ± 0.00 a	**
Estearic (C18:0)	2.32 ± 0.04 b	2.43 ± 0.13 b	2.43 ± 0.05 b	3.48 ± 0.01 a	3.55 ± 0.03 a	3.57 ± 0.02 a	***
cis-9-oleic (C18:1)	68.21 ± 1.42 a	66.98 ± 2.48 a	67.85 ± 0.79 a	60.41 ± 0.14 b	60.38 ± 0.26 b	60.68 ± 0.29 b	***
Linoléic (C18:2. Cis.cis)	11.74 ± 1.01 b	12.09 ± 0.27 b	12.28 ± 0.49 b	18.47 ± 0.17 a	19.03 ± 0.22 a	18.89 ± 0.2 a	***
linolenic (C18:3)	1.1 ± 0.04 a	1.12 ± 0.02 a	1.18 ± 0.09 a	0.99 ± 0.01 b	0.99 ± 0.01 b	0.97 ± 0.02 b	***
Araquic (C20:0)	0.38 ± 0.03 ab	0.41 ± 0.01 ab	0.36 ± 0.05 b	0.43 ± 0.00 a	0.42 ± 0.04 a	0.41 ± 0.01 ab	***
Gadoleic (C20:1)	0.19 ± 0.01 b	0.25 ± 0.01 a	0.25 ± 0.01 a	0.19 ± 0.01 b	0.20 ± 0.01 b	0.18 ± 0.01 b	***
*‘Picual’ cv.*							
Lauric (C12:0)	0.09 ± 0.01 b	0.11 ± 0.02 a	0.10 ± 0.01 ab	0.09 ± 0.01 b	0.07 ± 0.01 c	0.06 ± 0.00 c	***
Miristic (C14:0)	0.09 ± 0.01 a	0.10 ± 0.01 a	0.08 ± 0.01 a	0.09 ± 0.01 a	0.07 ± 0.00 b	0.06 ± 0.00 b	***
Palmitic (C16:0)	13.30 ± 0.19 a	12.78 ± 0.07 b	12.66 ± 0.13 b	11.22 ± 0.07 c	10.86 ± 0.08 d	10.89 ± 0.04 d	***
Palmitoleic (C16:1)	1.02 ± 0.04 a	0.94 ± 0.01 b	0.95 ± 0.02 b	1.05 ± 0.02 a	0.96 ± 0.01 b	0.96 ± 0.01 b	***
Margaric (C17:0)	0.04 ± 0.01	0.05 ± 0.00	0.03 ± 0.02	0.04 ± 0.00	0.04 ± 0.00	0.04 ± 0.00	ns
Margaroleic (C17:1)	0.05 ± 0.00 c	0.05 ± 0.00 bc	0.05 ± 0.00 c	0.07 ± 0.01 a	0.06 ± 0.00 b	0.06 ± 0.00 bc	***
Estearic (C18:0)	2.49 ± 0.08 c	2.67 ± 0.05 b	2.62 ± 0.04 b	3.10 ± 0.01 a	3.14 ± 0.02 a	3.13 ± 0.01 a	***
cis-9-oleic (C18:1)	74.53 ± 1.44	74.69 ± 0.42	75.30 ± 0.35	75.47 ± 0.31	75.55 ± 0.12	75.43 ± 0.10	ns
Linoléic (C18:2 cis,cis)	6.79 ± 1.2 bc	6.97 ± 0.35 abc	6.60 ± 0.30 c	7.41 ± 0.27 abc	7.84 ± 0.15 ab	7.99 ± 0.08 a	**
linolenic (C18:3)	1.02 ± 0.06 a	1.07 ± 0.03 a	1.02 ± 0.03 a	0.85 ± 0.02 b	0.84 ± 0.01 b	0.83 ± 0.01 b	***
Araquic (C20:0)	0.40 ± 0.05 ab	0.37 ± 0.01 abc	0.34 ± 0.01 c	0.40 ± 0.01 a	0.37 ± 0.01 abc	0.36 ± 0.00 bc	**
Gadoleic (C20:1)	0.17 ± 0.01 c	0.20 ± 0.01 a	0.18 ± 0.01 bc	0.21 ± 0.01 a	0.21 ± 0.00 a	0.19 ± 0.00 ab	***

Values expressed as percentage of total fatty acids. Means values followed by different letters indicate the existence of significant differences between samples by the Tukey test (*p* ≤ 0.05). Sig.: significance. ANOVA test significance levels: ns *p* > 0.05; ** *p* ≤ 0.01; *** *p* ≤ 0.001.

## Data Availability

The original contributions presented in this study are included in the article/Supplementary Materials. Further inquiries can be directed to the corresponding author.

## Supplementary Materials

The following supporting information can be downloaded at: https://www.mdpi.com/article/10.3390/foods15193549/s1. Figure S1. Representative HPLC-DAD/FLD chromatogram of the standard mixture used for the identification and quantification of phenolic compounds. (A) (255 nm) Oleuropein standard; (B) (320 nm) Verbascoside Standard; (C) (320 nm) standard mix; (D) (350 nm) standard mix; (E) Fluorescent standard mix. Figure S2. Representative high-performance liquid chromatography (HPLC) profiles of the olive pomace phenolic extracts. (A) Morisca green; (B) Morisca ripe; (C) Picual green; (D) Picual ripe. Figure S3. Representative Gas Chromatography (GC-FID) chromatogram of the standard mixture used for the identification and quantification of. Fatty Acid Methyl Esters (FAMEs) derived from the olive pomace. (A) Chromatogram of the standard mix (Supelco, 37 FAME mix); (B) table of calibration of peaks-FAME. Figure S4. Representative Gas Chromatography (GC-FID) chromatogram of the Fatty Acid Methyl Esters (FAMEs) derived from the olive pomace. (A) Morisca green; (B) Morisca ripe; (C) Picual green; (D) Picual ripe.

## Abbreviations

The following abbreviations are used in this manuscript:

ΔE	Color variation
ABTS	2,2′-azino-bis(3-ethylbenzothiazoline-6-sulfonic acid)
ANOVA	analysis of variance
CFU	Colony-forming units
DAD	Diode array detector
FAMEs	fatty acid methyl esters
FID	Flame ionization detector
FLD	Fluorescence detector
GAE	Gallic acid equivalent
HHP	High-Hydrostatic Pressure
MG	*Morisca* green
MR	*Morisca* ripe
PC1	Principal component 1
PC2	Principal component 2
PCA	Principal component analysis
PG	*Picual* green
PPO	Polyphenol oxidase
PR	*Picual* ripe
PVPP	polyvinylpyrrolidone
RI	Ripening index
RSM	Response Surface Methodology
TAA	Antioxidant activity
TPC	Total phenolic content
