# Supplementary material for: Effect of the Hydrostatic High Pressures (HHP) on the Microbiological and Physicochemical Properties of ‘Picual’ cv. and ‘Morisca’ cv. Olive Pomaces
Source: Foods. 2026 Oct 5;15(19):3549. doi: 10.3390/foods15193549 (PMC13664957; doi:10.3390/foods15193549)
Supplement: Supplementary file 1 [file foods-15-03549-s001.zip › foods-4538381-supplementary.pdf]

Supplementary Materials

Effect of the Hydrostatic High Pressures (HHP) on the microbiological and physicochemical properties of ‘*Picual*’ *cv.* and ‘*Morisca*’ *cv.* olive pomaces

Jesús García-Parra *, María Cabeza de Vaca, Manuel A. Martínez-Cañas and Antonia Trejo.

1. Phenolic Compounds Profile.

The phenolic extracts were analyzed using an Agilent 1200 HPLC system equipped with Diode Array (DAD) and Fluorescence (FLD) detectors. The detection parameters were tailored to the specific chemical nature of the target molecules. Highly purified commercial standards from Sigma-Aldrich and Extrasynthese were used to accurately identify each peak.

Figures S1. Representative HPLC-DAD/FLD chromatogram of the standard mixture used for the identification and quantification of phenolic compounds. (A) (255 nm) Oleuropein standard; B) (320 nm) Verbascoside Standard; C) (320 nm) standard mix; (D) (350 nm) standard mix; (E) Fluorescent standard mix.

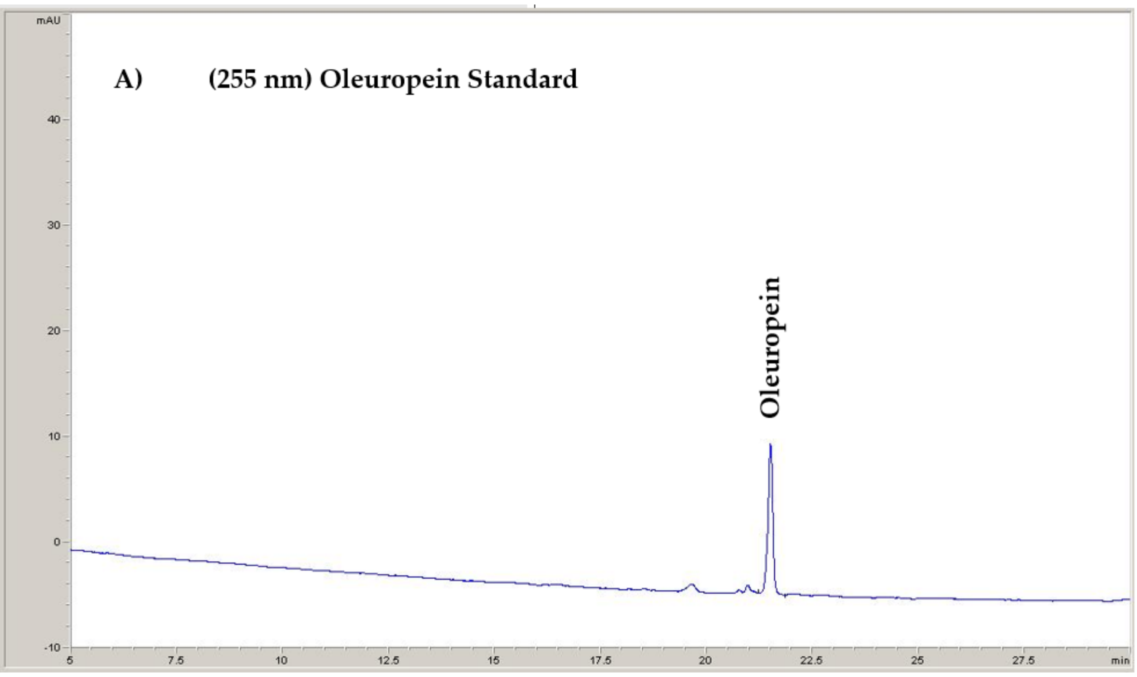

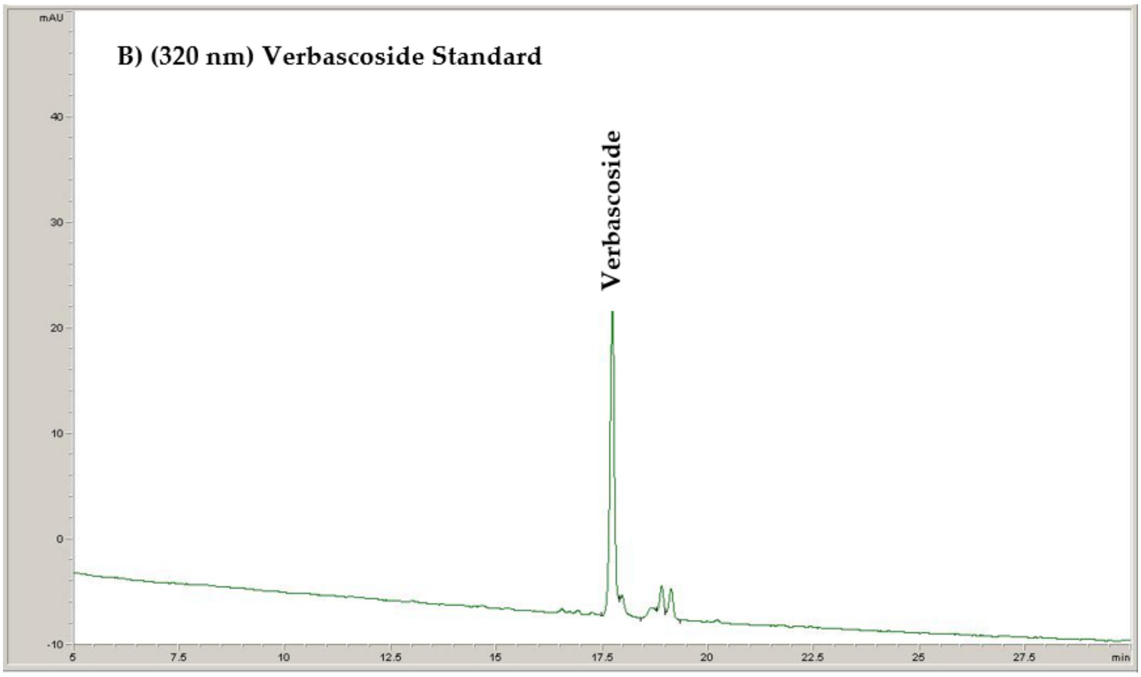

mAU
B) (320 nm) Verbascoside Standard
Verbascoside
40
30
20
10
0
-10
5
7.5
10
12.5
15
17.5
20
22.5
25
27.5
min

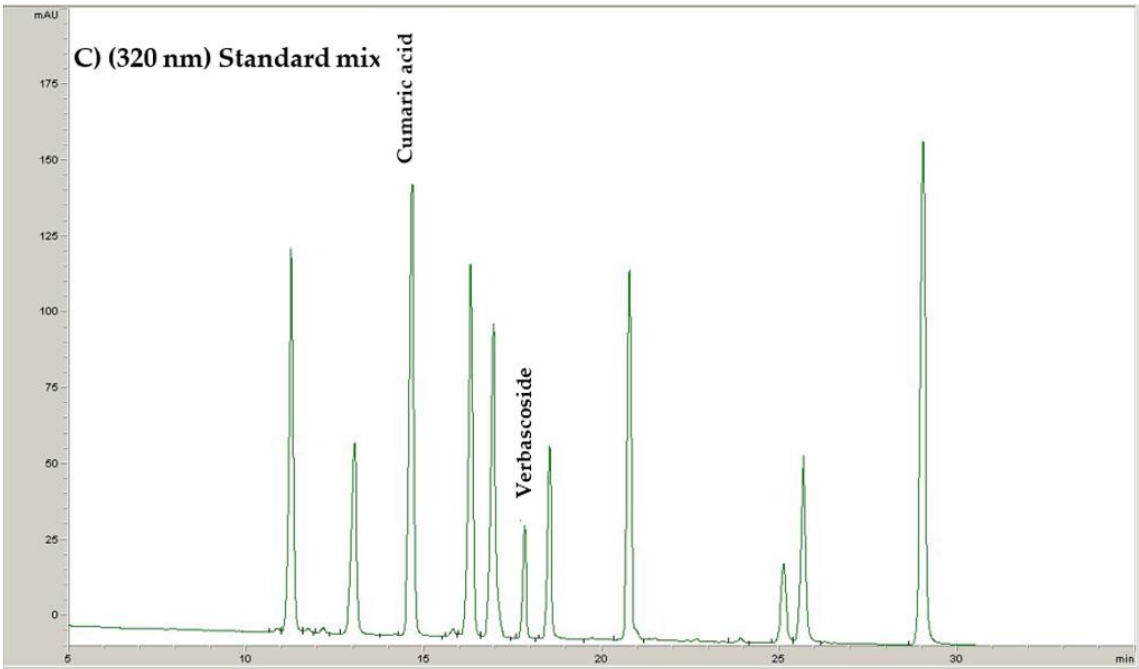

mAU
C) (320 nm) Standard mix
Cumaric acid
Verbascoside
175
150
125
100
75
50
25
0
5
10
15
20
25
30
min

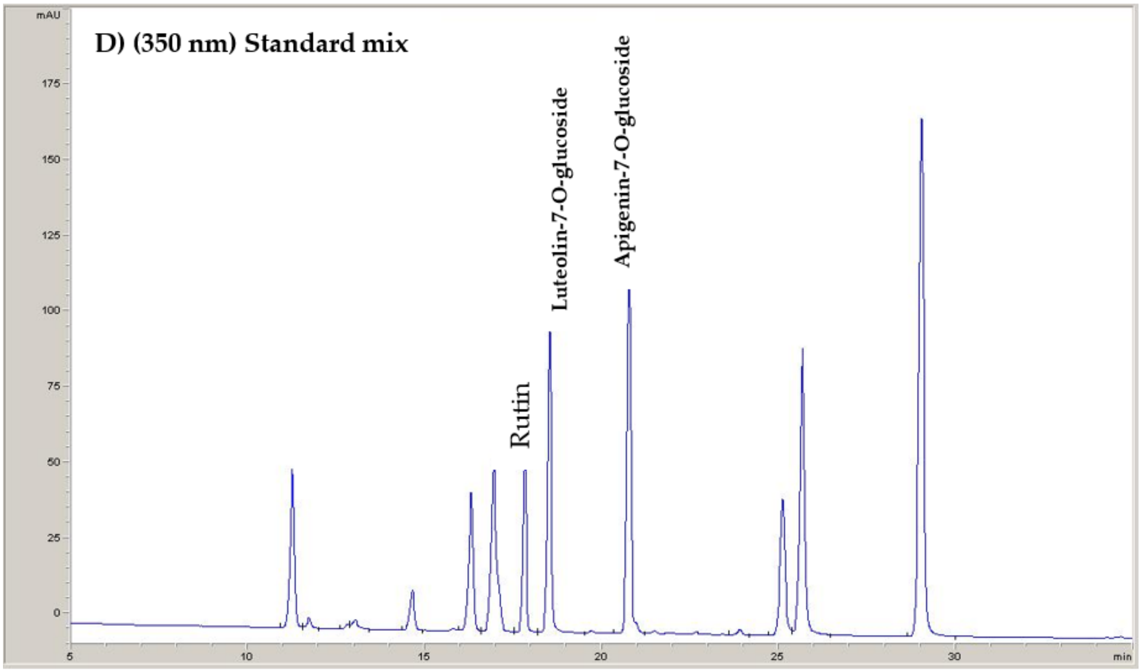
D) (350 nm) Standard mix
mAU
Rutin
Luteolin-7-O-glucoside
Apigenin-7-O-glucoside
min

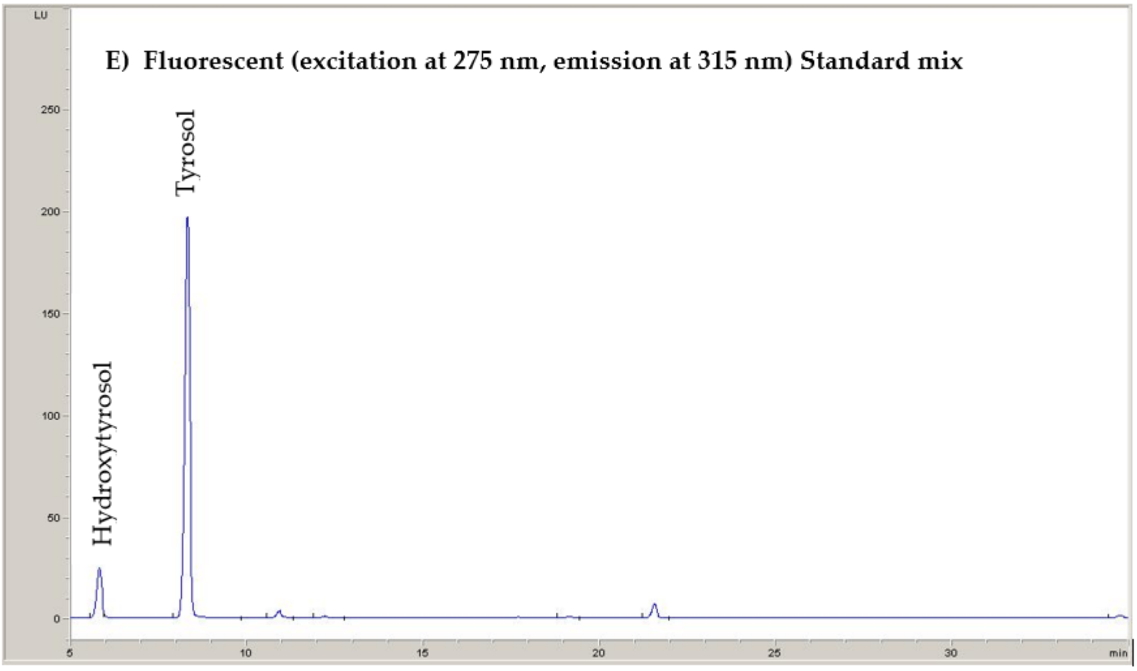
E) Fluorescent (excitation at 275 nm, emission at 315 nm) Standard mix
LU
Hydroxytyrosol
Tyrosol
min

Figures S2. **Representative high-performance liquid chromatography (HPLC) profiles of the olive pomace phenolic extracts. (A) Morisca green; (B) Morisca ripe; (C) Picual green; (D) Picual ripe.**

HPLC-FLD chromatogram (excitation at 275 nm and emission at 315 nm) demonstrating the specific detection, separation, and identification of key phenolic alcohols, highlighting the peaks for **hydroxytyrosol** and **tyrosol**. HPLC-DAD chromatogram monitored at 255 nm, illustrating the identification of the predominant secoiridoid, **oleuropein**. *(Note: Additional wavelengths at 320 nm and 350 nm were utilized for the identification of verbascoside, p-coumaric acid, and flavonoid glycosides through their respective authentic standards).*

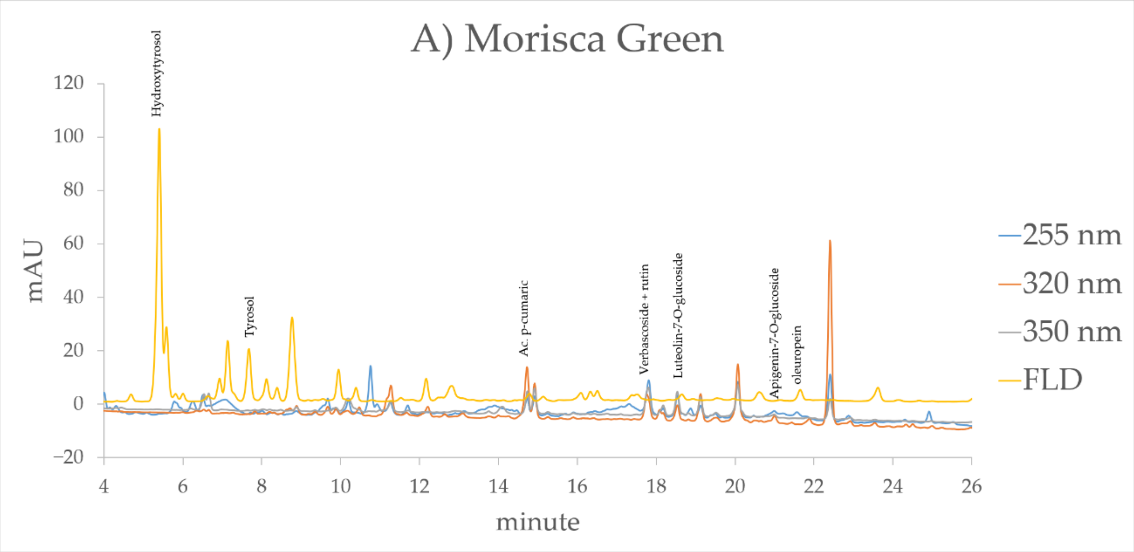

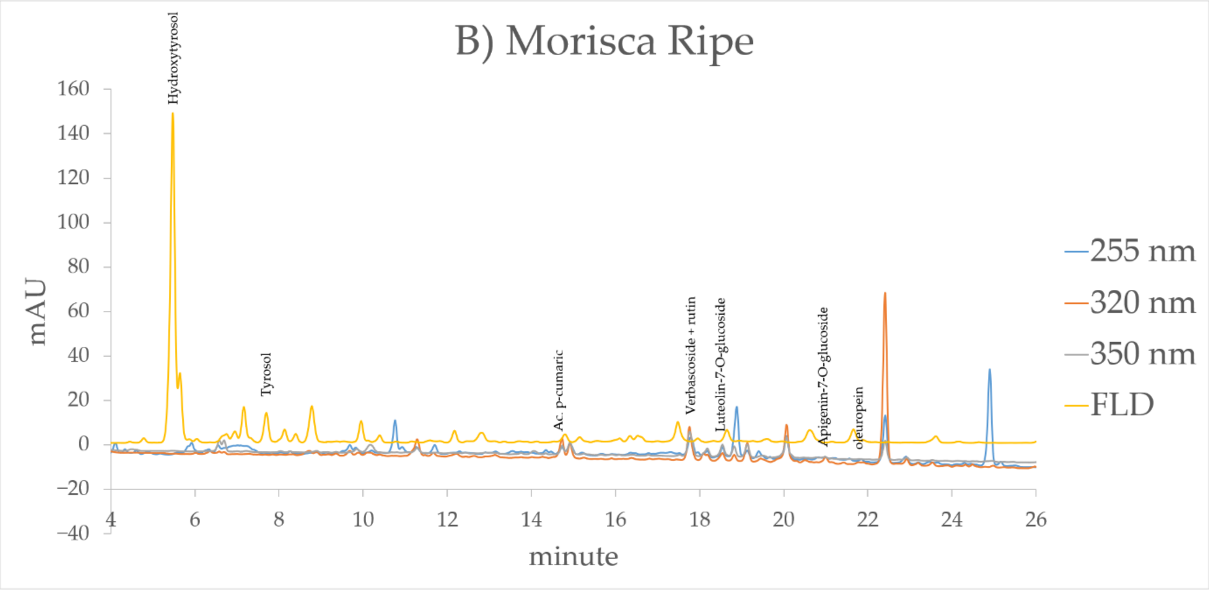

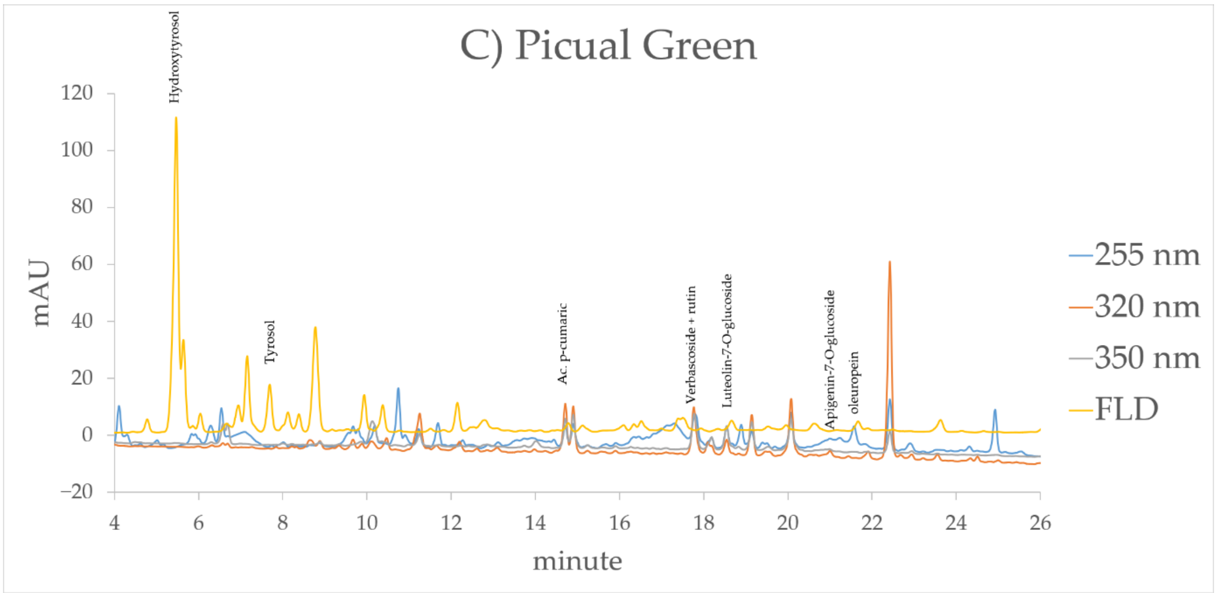
C) Picual Green
mAU
minute
Hydroxytyrosol
Tyrosol
Ac. p-cumaric
Verbascoside + rutin
Luteolin-7-O-glucoside
Apigenin-7-O-glucoside
oleuropein
255 nm
320 nm
350 nm
FLD

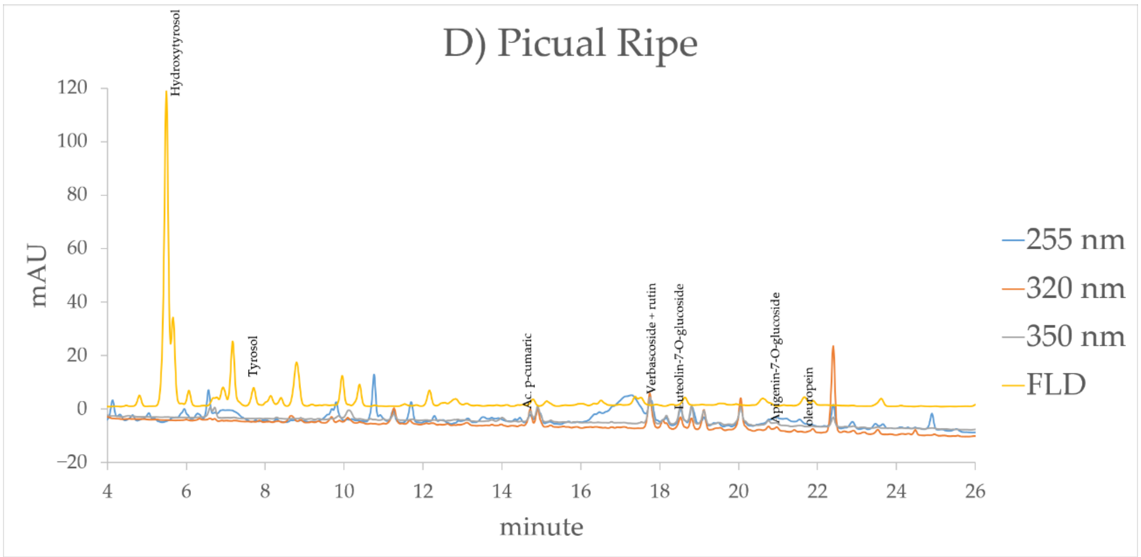
D) Picual Ripe
mAU
minute
Hydroxytyrosol
Tyrosol
Ac. p-cumaric
Verbascoside + rutin
Luteolin-7-O-glucoside
Apigenin-7-O-glucoside
oleuropein
255 nm
320 nm
350 nm
FLD

2. Fatty Acid Composition.

The lipid fraction was converted into Fatty Acid Methyl Esters (FAMEs) and analyzed using a Gas Chromatograph coupled with a Flame Ionization Detector (GC-FID, Agilent 6890) equipped with a DB-23 capillary column. The unequivocal identification of the fatty acids was confirmed using the commercial Supelco 37 Component FAME Mix standard (Sigma-Aldrich).

Figure S3. Representative Gas Chromatography (GC-FID) chromatogram of the standard mixture used for the identification and quantification of. Fatty Acid Methyl Esters (FAMEs) derived from the olive pomace. **A)** Chromatogram of the standard mix (Supelco, 37 FAME mix); **B)** table of calibration of peaks-FAME.

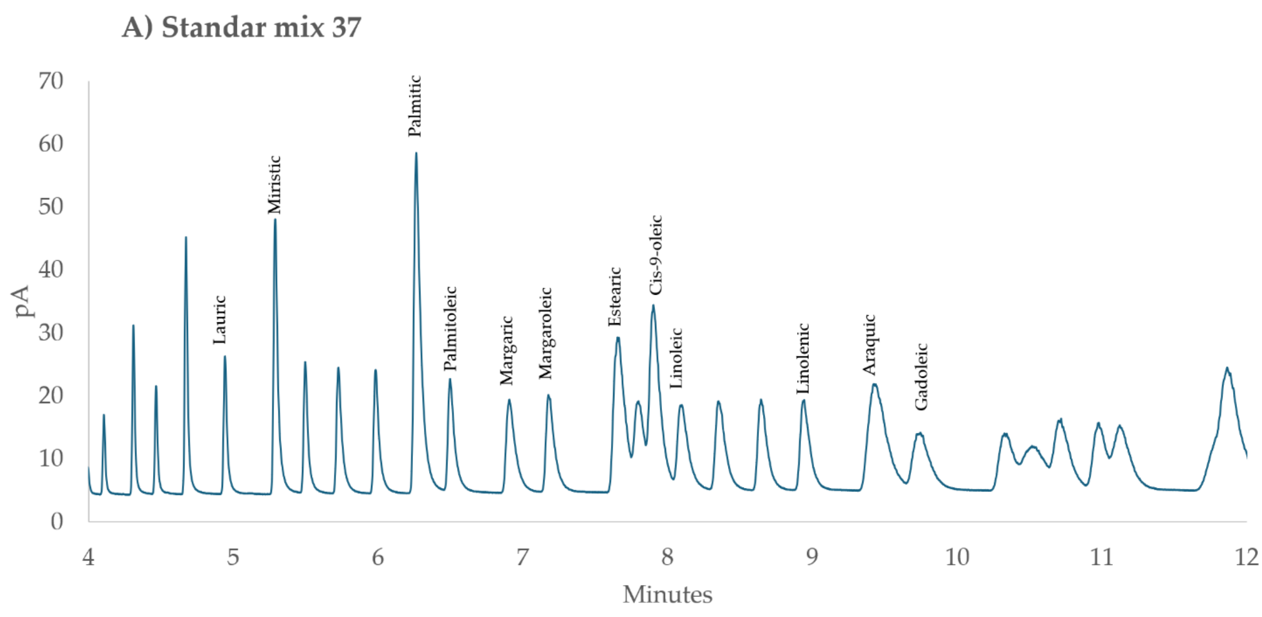

B) Calibration table of peaks detected in olive pomace

Calibration Table

Enter | Delete | Insert... | Print | OK | Help

#	RT	Signal	Compound	Lvl	Amt[% Area]	Area	Rsp.Factor	Ref	ISTD	#
1	4.974	FID1 A	Laurico (C12:00)	3	200.000	106.840	1.872	No	No	
2	5.330	FID1 A	Mirístico (C14:0)	3	400.000	232.290	1.722	Yes	No	
3	6.327	FID1 A	Palmitico (C16:0)	3	600.000	367.640	1.632	Yes	No	
4	6.564	FID1 A	Palmitoleico (C16:1	3	200.000	120.160	1.664	Yes	No	
5	6.981	FID1 A	Margárico (C17:0)	3	200.000	120.380	1.661	Yes	No	
6	7.258	FID1 A	Margaroleico (C17:	3	200.000	121.070	1.652	Yes	No	
7	7.749	FID1 A	Esteárico (C18:0)	3	400.000	224.000	1.786	Yes	No	
8	7.992	FID1 A	cis-9-oleico (C18:1	3	400.000	279.270	1.432	Yes	No	
9	8.455	FID1 A	Linoléico (C18:2, ci	3	200.000	121.260	1.649	Yes	No	
10	9.041	FID1 A	linolenico C18:3)	3	200.000	118.610	1.686	No	No	
11	9.549	FID1 A	Aráquico (C20:0)	3	400.000	240.030	1.666	Yes	No	
12	9.854	FID1 A	Gadoleico (C20:1)	3	200.000	122.470	1.633	Yes	No	
13	12.042	FID1 A	Erúcico (C22:1)	3	200.000	340.490	5.8739e-1	No	No	

Figure S4. Representative Gas Chromatography (GC-FID) chromatogram of the Fatty Acid Methyl Esters (FAMEs) derived from the olive pomace. **(A)** Morisca green; **(B)** Morisca ripe; **(C)** Picual green; **(D)** Picual ripe.

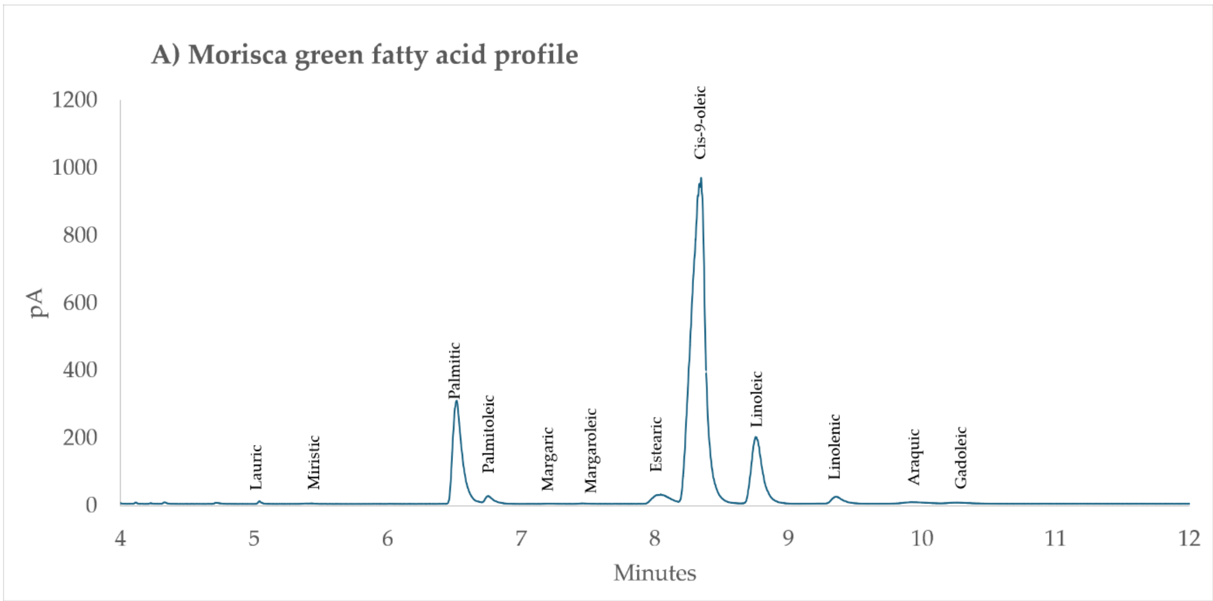

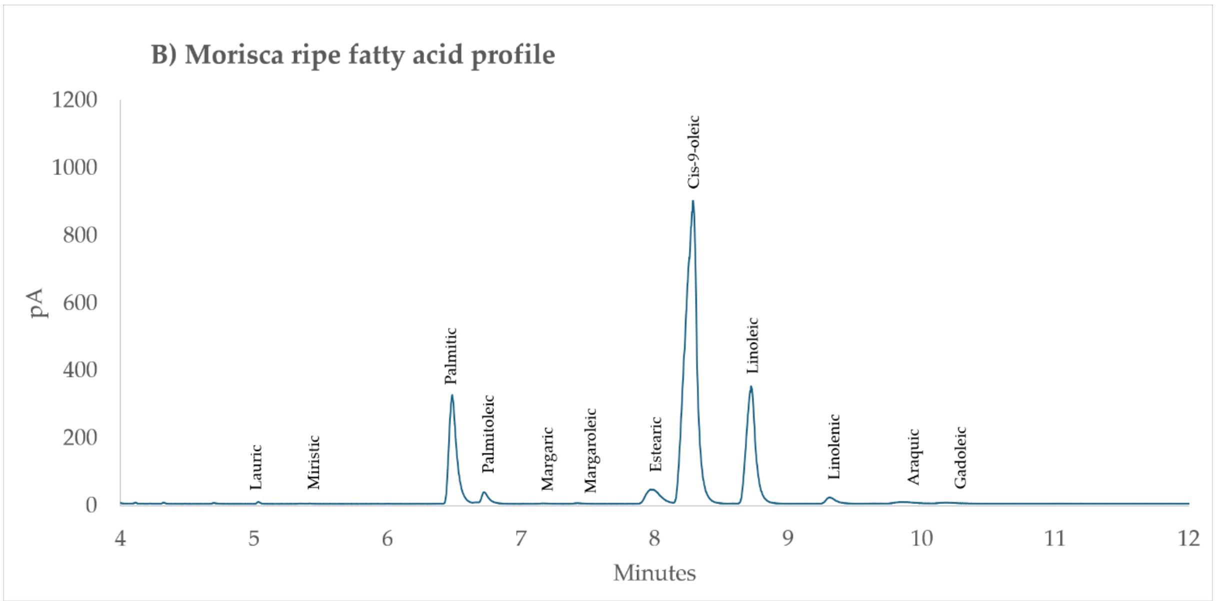

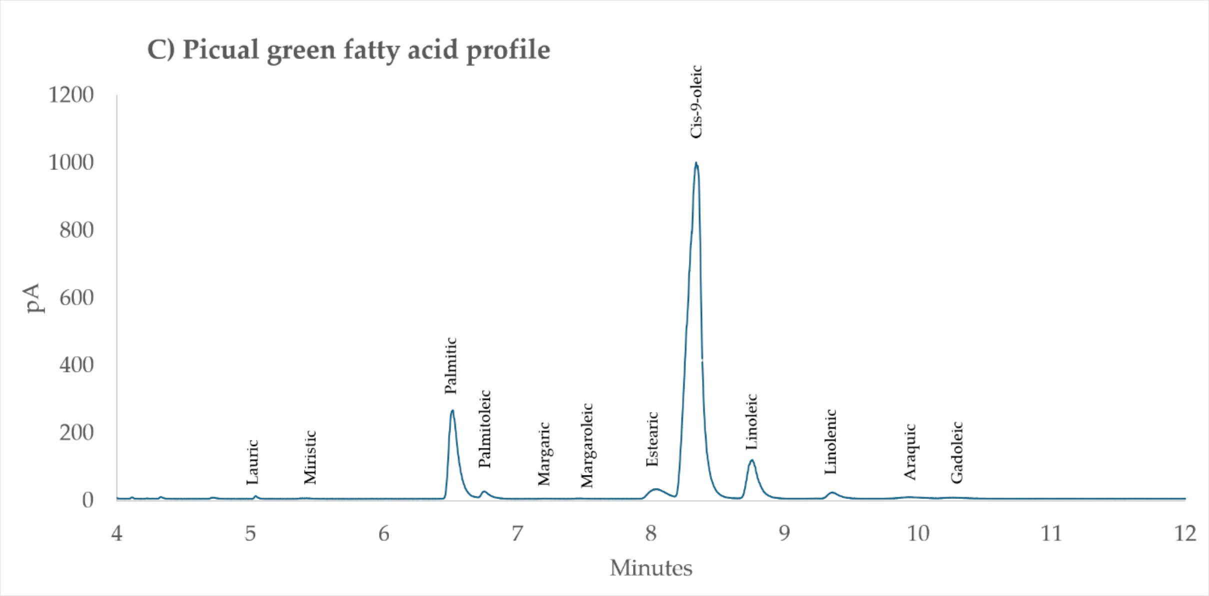
C) Picual green fatty acid profile
pA
1200
1000
800
600
400
200
0
4
5
6
7
8
9
10
11
12
Minutes
Lauric
Miristic
Palmitic
Palmitoleic
Margaric
Margaroleic
Estearic
Cis-9-oleic
Linoleic
Linolenic
Araquic
Gadoleic

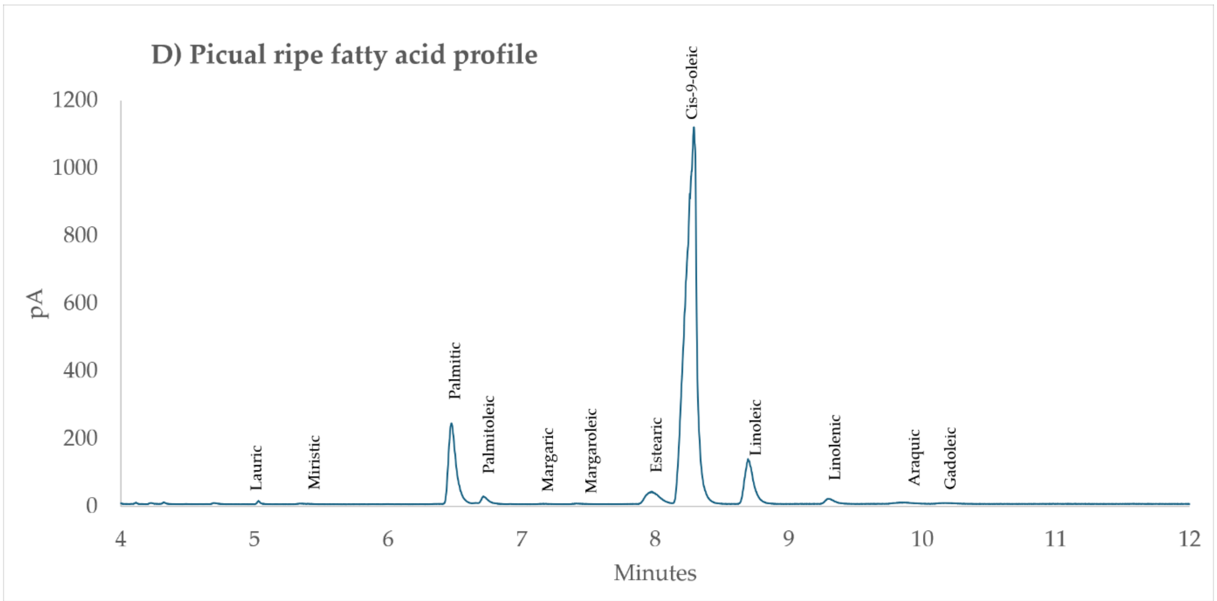
D) Picual ripe fatty acid profile
pA
1200
1000
800
600
400
200
0
4
5
6
7
8
9
10
11
12
Minutes
Lauric
Miristic
Palmitic
Palmitoleic
Margaric
Margaroleic
Estearic
Cis-9-oleic
Linoleic
Linolenic
Araquic
Gadoleic
